# Surrogate bias: Behavioral and physiological effects of seeing ingroup and outgroup virtual agents touching one's embodied avatar

**DOI:** 10.1016/j.chbr.2026.101273

**Published:** 2026-08

**Authors:** Matteo P. Lisi, Anna Giometti, Martina Fusaro, Salvatore M. Aglioti

**Affiliations:** a CLN2S@Sapienza, Fondazione Istituto Italiano di Tecnologia (IIT) and Dipartimento di Psicologia, Università La Sapienza, Rome, Italy; b LIFE Institute for Research and Health Care Santa Lucia IRCCS, Rome, Italy; c School of Advanced Studies, Centre for Neuroscience, University of Camerino, Camerino, Italy; d Consiglio Nazionale delle Ricerche (CNR), Istituto di Scienze e Tecnologie della Cognizione, Rome, Italy

## Abstract

Social touch is a primary channel for intergroup communication, yet how touch responses are shaped by group membership and individual differences (intergroup disgust, perceived attractiveness, ideological attitudes, implicit bias, and interpersonal distance preferences) remains poorly understood. Immersive virtual reality (IVR) offers a powerful tool to examine such processes under quasi-ecological yet controlled conditions. We recorded subjective and physiological responses — skin conductance and heart rate, used as autonomic arousal indices of implicit reactivity to socially salient stimuli — from heterosexual Caucasian participants (48 men, Experiment 1; 48 women, Experiment 2) as they observed touches applied to intimate, neutral, and social body areas of their gender-matched embodied avatar, delivered by Caucasian or African, male or female virtual agents. Behavioral results showed that men rated female avatars as more attractive, and attractiveness strongly predicted touch evaluations. Intergroup disgust predicted lower attractiveness of outgroup female avatars and greater interpersonal distance, while right-wing authoritarianism predicted greater touch appropriateness from female than male avatars. Women rated outgroup male avatars as more attractive; attractiveness predicted touch appropriateness, pleasantness, disgust, and erogeneity. Implicit racial bias indirectly reduced outgroup touch pleasantness via its effect on avatar attractiveness. Physiologically, men showed increased skin conductance to outgroup male touches on intimate and social areas, while women exhibited heart rate deceleration to intimate versus neutral touches. Interpersonal distance correlated with touch evaluation, suggesting a preparatory role for proxemics in social touch. Together, these findings demonstrate how IVR and physiological approaches can elucidate individual and social factors shaping intergroup proxemics, with implications for prejudice-reduction interventions.

## Highlights

• Psychophysiological reactivity may be similar for virtual and real social touch.
• Individual and relational variables shape intergroup virtual touch.
• Heterosexual, caucasian men and women rate as more attractive different-gender avatars.
• Intergroup disgust alters proxemics leading to increased inter-avatar distance.
• Implicit racial bias reduced the pleasantness of outgroup touch.

## 1 Introduction

Touch is a powerful channel of emotional communication, conveying a broad spectrum of emotions—including anger, fear, happiness, sadness, disgust, love, gratitude, and sympathy—through variations in pressure, speed, position, and movement (Hertenstein et al., 2009). Among these parameters, speed plays a critical role: stroking at velocities between 3 and 10 cm/s is associated with higher perceived pleasantness, a range that aligns with the activation profile of C-tactile (CT) afferents in the skin—slow-conducting, unmyelinated fibers thought to be specifically tuned to socially affective contact (Ackerley et al., 2014; Löken et al., 2009; Olausson et al., 2010). However, the meaning and impact of touch are determined not only by the mechanical properties of the stimulus (Ellingsen et al., 2016) but also by the social characteristics of the interactors (Gazzola et al., 2012; Mello et al., 2024, 2025) that may add to the experience of affective touch pleasure, relief, connection, discomfort, disgust, and avoidance. Gender plays a relevant role in tactile interactions, with women being more likely to value affective tactile stimulation and to exhibit greater tactile acuity (Russo et al., 2020). Furthermore, it appears that throughout life women experience touch more positively than men, feel more comfortable about touch, and engage in more frequent tactile interactions (Sorokowska et al., 2021; Trotter et al., 2018; Webb & Peck, 2015), possibly due to socio-cultural (Croy et al., 2019) and biological (Russo et al., 2020) factors. Relational context matters as well: there are implicit rules about which areas of the body are considered appropriate for touch and under which circumstances (Suvilehto et al., 2023), and closer emotional bonds allow access to more areas of the body and make touch more pleasurable (Suvilehto et al., 2015, 2019).

### 1.1 The role of group membership in tactile interactions

Humans automatically categorize others as belonging either to their own group (ingroup) or to another group (outgroup), a process considered a primary antecedent of intergroup bias and favoritism (Amodio & Cikara, 2021). Ingroup-outgroup categorization often relies on body-related visual information, such as physical traits associated with ethnic groups (Serino et al., 2009), with racial categorization remaining among the most pervasive ones (Amodio, 2014). Individual differences in dimensions such as right-wing authoritarianism, social dominance orientation, and disgust sensitivity further modulate how people respond in intergroup contexts (Bret et al., 2017; Kteily et al., 2011; Thomsen et al., 2008).

Despite the centrality of touch in social interactions, research on how racial group membership shapes tactile interactions remains scarce. The limited existing literature has mostly focused on the frequency and physiological impact of cross-race touch. Observational work among U.S. college students showed that cross-race touch was less frequent than same-race touch (Willis et al., 1978). Higher physiological arousal during outgroup touch was also found in a previous study, in which a same-sex Black nurse, compared with a same-sex White nurse, holding the participant's wrist for about 30 s elicited an increased heart rate in Caucasian American participants (Vrana & Rollock, 1998). Other studies have explored how physical contact, whether real or imagined, can reduce negative attitudes towards outgroup members. For example, a simple touch on the shoulder by a black experimenter led white participants to express more positive explicit and implicit attitudes towards black people (Seger et al., 2014). Similarly, imagining touching the hand of a black individual reduced both explicit and implicit biases towards the outgroup (Shamloo, Carnaghi, Piccoli, et al., 2018), an effect that was modulated by the positive appraisal of the intergroup contact (Shamloo, Carnaghi, & Fantoni, 2018). Together, these findings establish that intergroup touch is crucial to positive intergroup relations — yet the individual and relational factors that determine how outgroup touch is evaluated in the moment remain poorly understood.

Physiological measures offer a valuable complement to self-report in intergroup research, precisely because they are less susceptible to impression management and social desirability biases that may suppress explicit expressions of prejudice (Vanman et al., 1997). Skin conductance responses (SCRs) reflect sympathetic activation and are elevated for threatening and emotionally salient stimuli (Dawson et al., 2007), while heart rate (HR) changes dissociate two distinct response profiles: acceleration indexes sympathetic mobilization, whereas deceleration reflects parasympathetic orienting, attentional engagement, or freezing (Porges, 2009). Within the touch literature, affective CT-afferent mechanical stimulation delivered at optimal stroking velocities has been associated with HR deceleration and reduced SCRs relative to non-optimal touch (Pawling et al., 2017). Whether the racial group membership of the toucher produces comparable or additive effects on autonomic reactivity, and how such implicit physiological responses relate to explicit evaluations, are open questions the present study is designed to address.

### 1.2 Touch in immersive virtual reality

Most of the above studies on the effect of touch on intergroup dynamics use only self-report measures or may suffer from the methodological constraint of involving confederates. One possible solution to these limitations is offered by immersive virtual reality (IVR), which allows social interactions to be studied in controlled but ecologically valid environments (Blascovich et al., 2002; Yaremych & Persky, 2019). Perceptual and motor responses in virtual space activate neural mechanisms similar to those in the real world (Slater, 2009). Evidence shows that observing a virtual body from a first-person perspective (1 PP) generates a feeling of ownership (the sense that our body belongs to us; Ehrsson, 2020) over the virtual body (Maselli & Slater, 2013). Moreover, the mere observation of virtual caress on an embodied virtual body can induce vicarious sensations of being touched (Fusaro et al., 2016, 2019; Lisi et al., 2024; Nicolardi et al., 2025; Verga et al., 2025) and it can trigger maximal sexual arousal when touches are delivered to the virtual body's intimate areas by a touching avatar that matches the participant's sexual preference (Fusaro et al., 2021).

Additionally, touch evaluation in virtual environments might be anticipated by subjective preferences in the space that individuals maintain between themselves and others (Hall, 1966; Sorokowska et al., 2017). In IVR environments, interpersonal distance (IPD) has been used as a behavioral measure to investigate social comfort and tendencies of approach and avoidance (Bailenson et al., 2003; Fusaro et al., 2023; Lisi et al., 2021, 2026). Studies indicate that people tend to maintain a greater distance from avatars that belong to a different ethnic group (Dotsch & Wigboldus, 2008; Menshikova et al., 2018). This greater distance was associated with greater physiological activation (e.g., increased skin conductance) and levels of implicit bias, as measured by the Implicit Association Test (Dotsch & Wigboldus, 2008). In addition, interpersonal distance appears to predict subsequent behaviors toward the same avatars, such as increased aggression or decreased cooperation (McCall et al., 2009). Since physical contact requires bringing people closer together, the willingness to reduce physical distance from another person is a preliminary step to tactile interaction. Therefore, understanding interpersonal distance regulation in intergroup contexts can help clarify the mechanisms underlying the propensity or avoidance of physical contact with outgroup members.

### 1.3 The present study

Building on studies about the similar reactivity to real and virtual touch (Fusaro et al., 2016, 2019, 2021; Mello et al., 2022; Nicolardi et al., 2025; Verga et al., 2025), we created a novel virtual reality paradigm for probing explicit (behavioral) and implicit (psychophysiological) responses to observation of Caucasian and African artificial virtual agents touching the embodied avatar of Caucasian heterosexual men (Experiment 1) and Caucasian heterosexual women (Experiment 2). This approach allowed us to examine preferences for ingroup touch across varying levels of implicit racial prejudice and gender, while also exploring the possible dissociation between the explicit and implicit attitudes. Given the central role of specific body regions in shaping the effect of touch (Fusaro et al., 2021; Mello et al., 2022, 2024), we also explored differences in the reactivity to stimuli delivered in intimate, social, and neutral body regions. Furthermore, since racial prejudice may interact with gender (Sidanius et al., 2018), we presented both female and male avatars to explore this possible interaction.

### 1.4 Hypotheses of the present study

Based on previous evidence that touch perception is shaped by both the toucher's gender and group membership (Saarinen et al., 2021; Suvilehto et al., 2023), we expected that, in both women and men, touches from female avatars and avatars from their own ethnic group would be evaluated more positively. Furthermore, we explored whether higher levels of implicit bias, as measured by the Implicit Association Test and trait questionnaires related to authoritarianism and social dominance orientation, would be associated with more negative subjective evaluations of touches administered by avatars of African ethnicity. We also predicted that such touches might evoke more pronounced physiological responses, such as increased skin conductance responses and heart rate acceleration, the latter being consistent with previous findings on intergroup touch (Vrana & Rollock, 1998). However, we acknowledged that heart rate responses to intergroup touch may be twofold: while acceleration would reflect sympathetic mobilization and aversive arousal, deceleration could alternatively index parasympathetic orienting — an attentional engagement response to a novel or socially salient stimulus (Bradley et al., 2001; Mello et al., 2022). Finally, we expected IPD to follow a similar pattern to touch evaluations, with greater distance maintained toward outgroup avatars (Dotsch & Wigboldus, 2008), and we explored its correlation with touch reactions-specifically lower pleasantness and appropriateness and higher disgust-based on the notion that individuals less comfortable with physical proximity to outgroup members should also respond more negatively to their touch.

## 2 Materials and methods

### 2.1 Participants

A power analysis for the repeated-measures ANOVA testing the three-way interaction between Touching Avatar Gender, Touching Avatar Ethnicity, and Area with (α = .05, power = 0.80, Cohen's F = 0.325, based on a previous study using a similar paradigm; Fusaro et al., 2021), conducted with MorePower 6.0 (Campbell & Thompson, 2012), indicated a minimum of 48 participants. 48 heterosexual Caucasian men (Experiment 1; mean age = 26.5, SD = 5.20) and 48 heterosexual Caucasian women (Experiment 2; mean age = 24.1, SD = 2.94) with normal or corrected visual acuity and naïve as to the purposes of the study were recruited (for complete demographics, see Table 1). Before taking part in the study, participants rated their sexual orientation on a Kinsey scale ranging from “exclusively heterosexual” (0) to “exclusively homosexual” (100), with “bisexual” (50) in the middle of the VAS (mean ± SD in the Kinsey scale M = 4.00 ± 7.73, F = 5.54 ± 8.24). The VAS range was divided by the Kinsey scale's seven categories: 0–14.28 = exclusively heterosexual; 14.28–28.57 = predominantly heterosexual, only incidentally homosexual; 28.57– 42.85 = predominantly heterosexual, but more than incidentally homosexual; 42.85–57.14 = equally heterosexual and homosexual; 57.14–71.42 = predominantly homosexual but more than incidentally heterosexual; 71.42– 85.71 = predominantly homosexual, only incidentally heterosexual; 85.71–100 = exclusively homosexual. The cutoff to be included in our sample was 28.57. The experimental protocol was approved by the ethics committee of the Fondazione Santa Lucia (CE/2022_012) and was carried out in accordance with the ethical standards of the 2013 Declaration of Helsinki. All participants gave their written informed consent to take part in the study and did not receive compensation. In a single session, they completed the virtual touch task, gave post-touch ratings (ownership, vicarious touch, avatar attractiveness, trustworthiness), performed the interpersonal distance task and the IAT, and then completed the questionnaires.

**Table 1:** Demographic characteristics of the Caucasian heterosexual men (Experiment 1) and the women (Experiment 2).

Sex	N	Age	Sexual Orientation	Political Orientation	Touch Avoidance	Social Dominance Orientation	Right Wing Authoritarianism	Intergroup Disgust	Implicit Racial Bias
M	48	26.5 ± 5.20	4.00 ± 7.73	4 = right;	29.87 ± 10.31	2.65 ± 0.97	2.88 ± 1.05	2.12 ± 0.85	0.43 ± 0.42
				12 = center-right;					
				17 = center-left;					
				15 = left;					
F	48	24.1 ± 2.94	5.54 ± 8.24	1 = right;	31.85 ± 10.89	2.19 ± 0.80	2.55 ± 0.66	1.82 ± 0.85	0.27 ± 0.37
				11 = center-right;					
				15 = center-left;					
				21 = left;					

### 2.2 Experimental stimuli and setup

The virtual scenario was designed using 3DS Max 2017 (https://www.autodesk.com/it/products/3ds-max/overview) and the virtual avatars were created using Character Creator 3 (https://www.reallusion.com/character-creator/) and implemented in Unity 5.6 (https://unity.com/). The avatars were validated for their levels of Eurocentricity and Afrocentricity (D. S. Ma et al., 2015), perceived gender, and age (see Table 2). Caucasian avatars received mean Eurocentricity ratings above 4 on the 5-point Likert scale, while African avatars received mean Afrocentricity ratings above 4, confirming that each avatar was successfully perceived as belonging to the intended ethnic group.

**Table 2:** Avatar stimulus validation.

Avatar	Age	Gender	Afrocentrism	Eurocentrism
Ingroup Female	28.7 ± 4.2	Female = 100%	1.3 ± 0.5	4.3 ± 0.7
Ingroup Male	31.1 ± 4.1	Male = 100%	1.3 ± 0.5	4.1 ± 0.8
Outgroup Female	24.4 ± 5.3	Female = 96%	4.7 ± 0.5	1.4 ± 0.6
Outgroup Male	25.8 ± 4.3	Male = 96%	4.4 ± 0.6	1.7 ± 0.7

*Note*. Ratings were provided by 81 Caucasian respondents aged 18-40 years. Afrocentrism and Eurocentrism were evaluated on a 5-point Likert scale (1 = *not at all*, 5 = *totally*). Gender was assessed via binary choice. Age was estimated in years.

The scenario was presented through the Meta Quest 2 (https://www.meta.com/) head-mounted display (HMD). Touch animations were reused from our previous studies (Fusaro et al., 2021; Mello et al., 2022), where an actor gently caressed different body parts of another actor seated on a beach chair using the right hand, at an instructed speed of approximately 3 cm/s — a velocity within the range optimal for C-tactile afferent activation. Kinematics were recorded using an Xsens motion capture suit and transferred onto the virtual avatars for rendering in Unity. Touch was visual only, and no haptic input was provided.

**Fig. 1:**
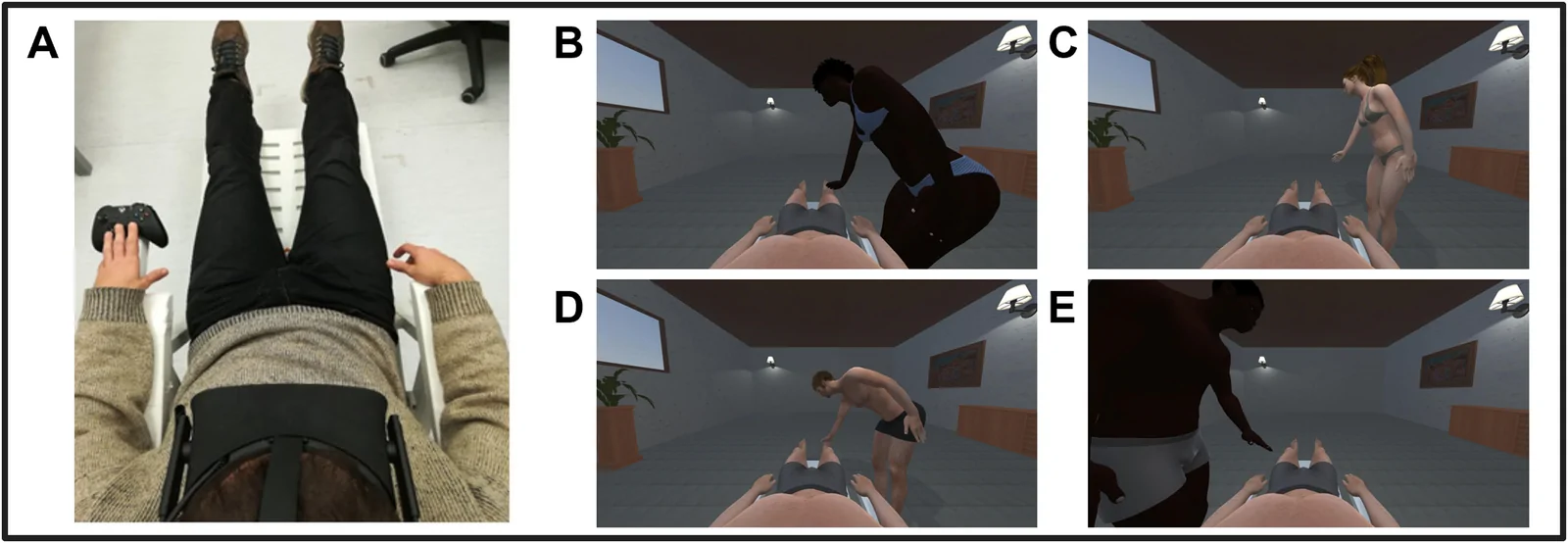
Experiment 1. Caucasian heterosexual men observed a gender-matched virtual body from a first-person perspective (A). The virtual touch task was divided into four blocks. In each block, the 1 PP virtual body was caressed either by a dark-skinned female avatar (B), a light-skinned female avatar (C), a light-skinned male avatar (D), or a dark-skinned male avatar (E).

**Fig. 2:**
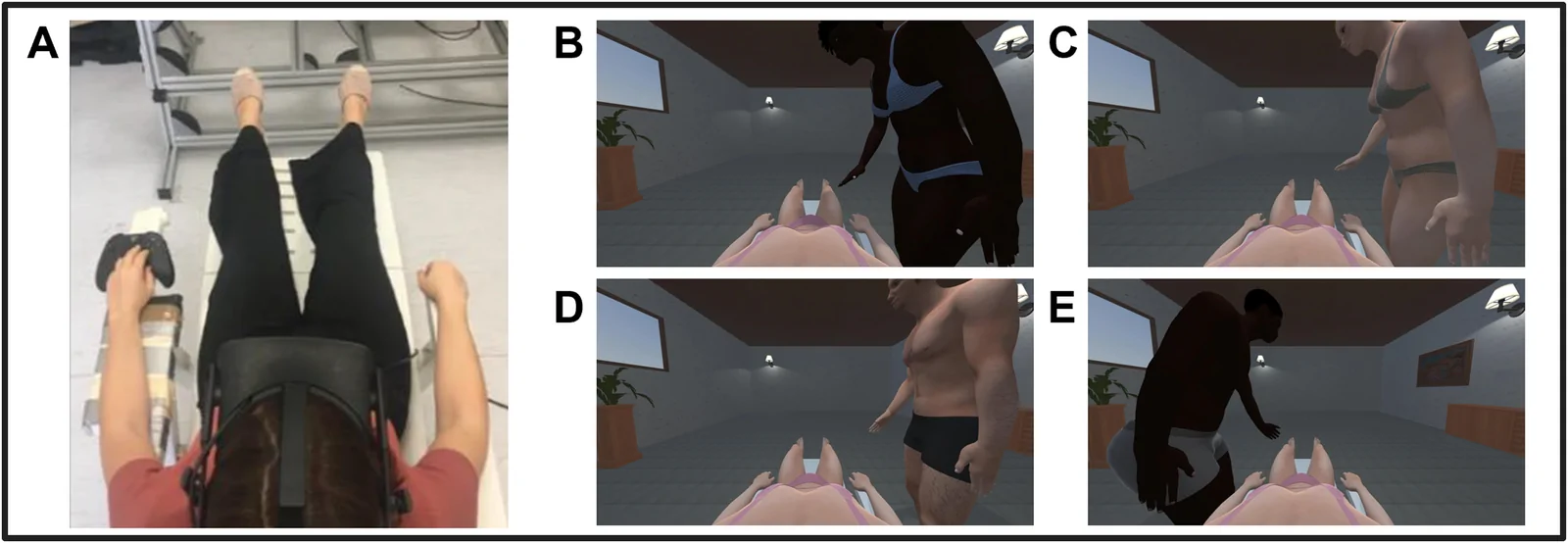
Experiment 2. Caucasian heterosexual women observed a gender-matched virtual body from a first-person perspective (A). The virtual touch task was divided into four blocks. In each block, the 1 PP virtual body was caressed either by a dark-skinned female avatar (B), a light-skinned female avatar (C), a light-skinned male avatar (D), or a dark-skinned male avatar (E).

The reclining beach-chair posture was chosen to provide a standardized and controllable setting in which multiple body regions were visible and reachable while minimizing differences in posture, movement, interpersonal distance, and occlusion across trials. Although not intended to reproduce a typical everyday interaction, this scenario allowed us to compare responses to touch across body parts and avatar categories under controlled conditions. Moreover, we presented the avatars in underwear (Fig. 1, Fig. 2) to increase the salience of the touched body regions while reducing clothing-related social cues, such as social status or group affiliation (Świdrak et al., 2021).

### 2.3 Virtual touch task: general procedure

All participants were seated on a beach chair and wore an HMD through which they observed a gender-congruent virtual body from a 1 PP (Fig. 1A). The experiment was composed of four separate blocks presented in a randomized order. In each block, participants observed the touches delivered on their gender-congruent virtual body by either a light- or dark-skinned male avatar (Fig. 1D and E, respectively) or a light- or dark-skinned female avatar (Fig. 1C and B, respectively; see the video in the Supplementary Information). Each block consisted of 12 touches (in 6 trials the avatar was on the left and in 6 on the right). The body parts touched by the avatar were: foot and knee (Neutral areas), genitals and breast (Intimate areas), head and hand (Social areas); these body parts were categorized in Neutral, Intimate, and Social areas for the purpose of the statistical analyses based on a previous validation (Fusaro et al., 2021). Each trial started with the observation of the participant's own virtual body. Then the touching avatar appeared and, after about 1 s, caressed a given body part. The touch lasted for ∼3 s at a speed of ∼3 cm/s. Participants continued to observe their own virtual body for 7000 ± 500 ms after the stimulus and, at the end of the trial (and the end of the observation), the first of four visual analogue scales (VAS) appeared on a screen in the virtual environment, always in the same fixed order. The VAS assessed Appropriateness, Pleasantness, Disgust and Erogeneity (see Table S1). Participants were asked to rate the sensations elicited by the observation of the stimuli on a scale from 0 to 100. During the experiment, galvanic skin response and ECG signals were continuously recorded. All participants were requested to look at their virtual body throughout the experiment and focus their attention on the toucher's hand during touch. After each stimulus presentation, a black panel appeared in the virtual scene in front of the virtual body. The panel displayed a horizontal green line (60 cm length) with the extremities signed by “−−” and “++”. Participants were instructed to use the left hand to use the controller that moved a vertical bar along the VAS line to provide their responses. At the end of each of the four blocks, participants were asked to answer, using an identical VAS, four questions about the embodiment over the virtual body, one question about the sensation elicited by the vicarious touches, and two questions about the attractiveness and trustworthiness of the touching avatar. To check that the task instructions were understood, before wearing the HMD, participants read a description of each VAS (see SI).

### 2.4 Physiological recording and pre-processing

Skin conductance response (SCR) and heart rate (HR) were used as measures of physiological reactivity. An ADInstruments PowerLab 8/35 device was used as a signal amplifier along with the ML116 GSR Amplifier (providing a 75 Hz AC excitation with a low constant voltage of 22 mVrms) with specific galvanic skin response sensors consisting of two bipolar finger electrodes. The sensors were applied on the distal phalanx of the index and middle fingers of the right hand, and the signal was sampled at 1 kHz. For the electrocardiography, two electrodes (DORMO pre-gelled electrodes, 50 mm) were placed on the back of each hand, and the reference was placed on the left ankle. Signals were sampled at 1 kHz and filtered using a 30-Hz low-pass filter. Data were recorded using the LabChart 7 software (ADInstruments, Inc.). Inter-beat intervals were computed and then converted into HR in beats per minute (bpm) using LabChart. Data were reduced offline in 1-s bins. HR changes contingent upon observation of virtual caresses were computed as differential values between 3 s after a virtual caress was delivered and 2 s of baseline before the appearance of the touching avatar. Trials with bad signal quality were removed (0% of the dataset in Experiment 1; 6.33% of the dataset in Experiment 2). Additionally, values exceeding ±2 standard deviations from the group mean were removed (5.08% of the dataset in Experiment 1; 3.82% of the dataset in Experiment 2). For SCR, the subtraction between the maximum and the minimum values (with a minimum response of 0.01 μS) in a time window of 6 s after the stimulus onset was computed. Additionally, we extracted skin conductance level (SCL) and Heart Rate Variability (HRV) indexes from the four blocks and the 1-min resting period (details and results are reported in the Supplementary Information).

### 2.5 Moderating psychological factors

#### 2.5.1 Social Dominance Orientation

Participants completed a 10-item Italian SDO scale (Di Stefano & Roccato, 2005) to assess their preference for social dominance hierarchy. SDO is defined as the degree to which a person generally supports a system of group-based hierarchy (Pratto et al., 1994). Participants responded on a scale ranging from 1 (do not agree at all) to 7 (strongly agree). Cronbach's α was 0.85 for men and 0.80 for women.

#### 2.5.2 Right Wing Authoritarianism

Participants completed the 10-item RWA scale (Manganelli Rattazzi et al., 2007) responding on a scale ranging from 1 (do not agree at all) to 7 (strongly agree). Cronbach's α was 0.84 for men and 0.77 for women. People high in authoritarianism exhibit high degrees of deference to established authority, aggression toward outgroups when authorities permit that aggression, and support for traditional values when those values are endorsed by authorities (Whitley Jr, 1999).

#### 2.5.3 Intergroup Disgust Sensitivity

Participants completed the 8-item Intergroup Disgust Sensitivity Scale (Hodson et al., 2013) translated into Italian. This questionnaire measures the negative affective reactions to social outgroups characterized by revulsion. Participants were asked to rate each sentence on a 7-point Likert scale ranging from strongly disagree to strongly agree. Cronbach's α was 0.77 for men and 0.79 for women.

#### 2.5.4 Social Touch Questionnaire

Participants completed the 20-item Social Touch Questionnaire (STQ; Wilhelm et al., 2001), translated into Italian. This questionnaire assesses social touch avoidance, with higher scores indicating greater levels of touch avoidance. Participants rated each statement on a 5-point Likert scale ranging from strongly disagree to strongly agree. Cronbach's α was = 0.84 for men and 0.86 for women.

#### 2.5.5 Race Implicit Association Test

We assessed implicit racial bias with a computerized version of the Race IAT (https://osf.io/jvq4q/) presented on a PC monitor using PsychoPy v1.90. IAT measures the ease and strength of automatic associations between pairs of target categories and evaluative categories (Greenwald et al., 2003). The stimuli consisted of pictures of the target categories (faces of White and Black people) and words indicating the evaluative categories (disgusting or pleasant; Greenwald et al., 2003). The category labels were presented on the left and right of the stimulus word and remained on the computer screen throughout each block. The categories were mapped onto two keys on a keyboard, with the “E” key representing the categories on the left half of the screen and the “I” key representing the categories on the right half of the screen. The IAT's order was randomized (i.e., “Disgusting- Black People” and “Pleasant- White People” response settings in blocks 3–4 and “Disgusting - White People” and “Pleasant - Black People” response setting in blocks 6–7) across participants (Greenwald et al., 2003). D scores were calculated according to the procedure described by Greenwald and colleagues (2003) and averaged to obtain a final IAT D score. We programmed the test so that positive D scores are obtained with pleasant-white pairing and disgusting-black pairing; in contrast, negative D scores are obtained with pleasant-black pairing and disgusting-white pairing. The final D score represents a relative measure of the implicit preference for a given ethnic group.

#### 2.5.6 Interpersonal distance task

The interpersonal distance task was administered after the main touch task (see Fig. S1 and supplementary video). Participants completed a passive version (standing still) of the task (Iachini et al., 2016). Prior to the task, participants completed a calibration phase to align the avatar's height with their own. Participants adjusted the avatar's proximity using the right controller of the Meta Quest headset. Instructions were as follows: “Keep the button “A” pressed to make the avatar walk toward you. Release the button to stop the avatar. Once the distance is comfortable for you, utter the word “stop”. The participant's vocal cue was used as the stopping point, and the experimenter recorded the distance by pressing a key on the keyboard. Participants completed four blocks (one per avatar) in randomized order, with three trials per block. The distance between the participant and the avatar was continuously calculated in Unity using two virtual reference points: the headset and the avatar's nose. The stopping distance (in cm) was recorded at the moment the avatar was stopped. For each avatar, the mean stopping distance across the three trials was computed. The avatars were those employed for the virtual touch task.

### 2.6 Data analysis

Linear mixed models were used to analyze each dependent variable namely, the scores for each of the four VAS questions (Appropriateness, Pleasantness, Disgust, Erogeneity), Interpersonal Distance, SCR (μS, in the 6 s post-stimulus time window), heart rate (HR, in the 3 s post-stimulus time window), and tonic physiologic arousal (skin conductance level and Heart Rate Variability measures (reported in the SI). All models included as fixed effects the main effects and interactions between experimental factors: avatar gender (male or female), avatar ethnicity (Caucasian or African), and body area (intimate, social, neutral) categorized on the basis of a validated procedure (Fusaro et al., 2021); see Fig. 1. In all models, participants were always set as a random effect, with correlated intercept and slopes for avatar gender, avatar ethnicity, and body area. A simpler random effect structure was set when the model failed to converge or showed a singular fit. For each of the dependent variables, we conducted a two-stage model comparison using Akaike's Information Criterion (Akaike, 1974; Cavanaugh & Neath, 2019) to test the effect of additional predictors. In the first stage, we tested the covariate effect of avatar attractiveness and trustworthiness, as well as participants' levels of social touch avoidance. In the second stage, the best-fitting model from the first stage was compared against extended models that included the moderating effects of social attitudes (SDO, RWA, IAT, Intergroup disgust sensitivity) on the interaction between touching avatar ethnicity and touching avatar gender. Additionally, we examined the relationships between touch appropriateness, pleasantness, disgust, erogeneity, interpersonal distance (IPD), skin conductance response (SCR), and heart rate (HR) using Pearson correlation analyses. We assessed correlation strength using the following criteria: r ≤ |0.12| — very weak correlation; |0.12| ≥ r ≥ |0.24| — weak correlation; |0.24| ≥ r ≥ |0.41| — moderate correlation; |0.41| ≥ r ≥ |1| — high correlation (Lovakov & Agadullina, 2021). P-values were FDR-corrected for multiple comparisons (Benjamini & ​Hochberg, 1995). Finally, for the ratings administered at the end of each block, two separate linear mixed models were constructed: one with ratings of embodiment as the dependent variable and question type (e.g., embodiment, control), touching avatar gender, and touching avatar ethnicity as fixed predictors; the other with ratings of vicarious touch as the outcome and avatar gender and ethnicity as fixed predictors. Post-hoc comparisons were performed using Tukey-corrected estimated marginal means (EMMs). The Supplementary Information (SI) provides the complete model syntax and reports additional results on avatar feature ratings, body ownership, vicarious touch, touch disgust, and tonic physiological arousal.

## 3 Experiment 1: heterosexual caucasian male participants

### 3.1 Results

#### 3.1.1 Virtual touch task

##### 3.1.1.1 Appropriateness of touch

**Fig. 3:**
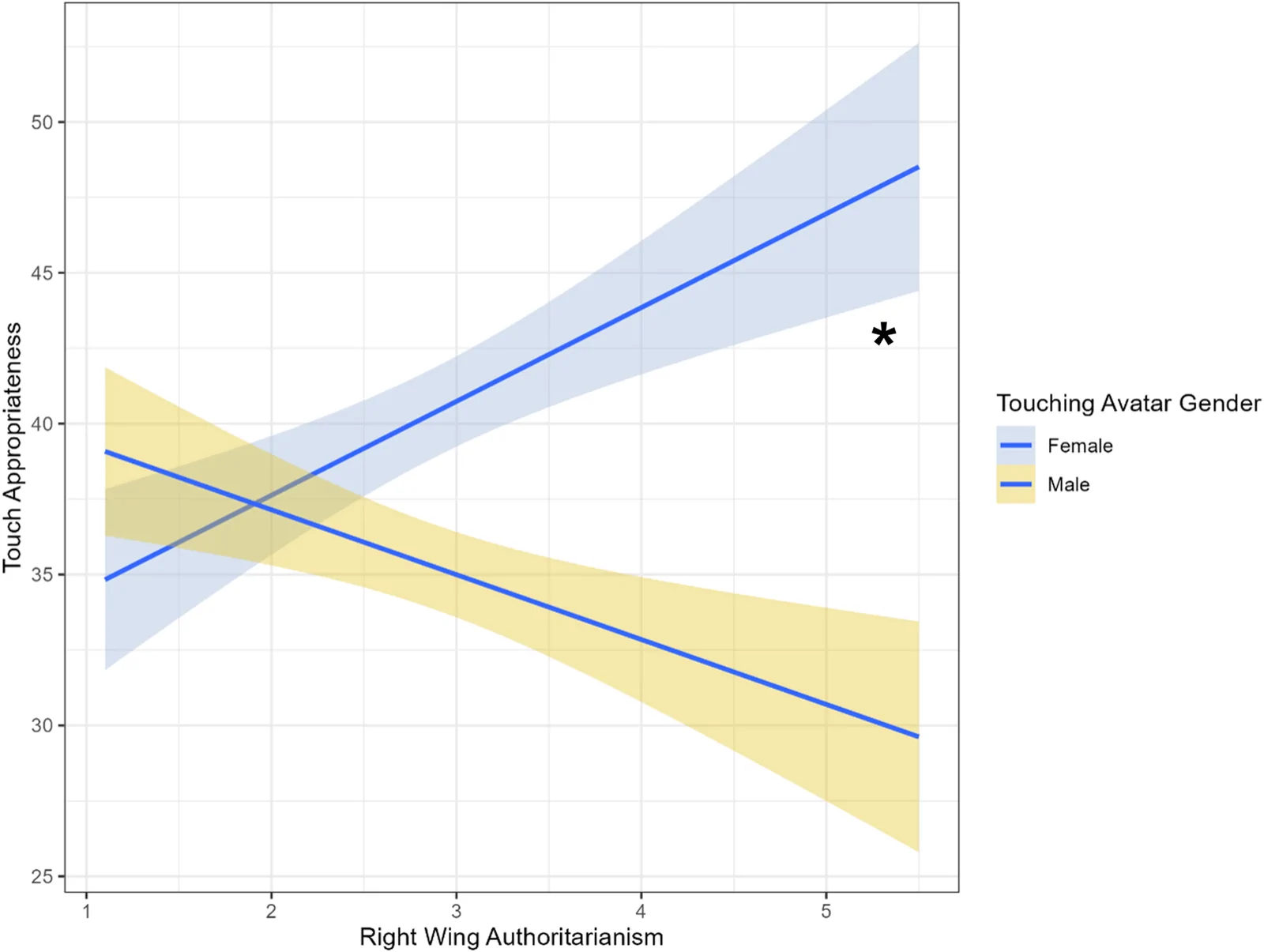
Caucasian heterosexual men (Experiment 1). Higher levels of Right Wing Authoritarianism–reflecting a stronger tendency to endorse traditional social conventions and submit to perceived authority–were associated with increased appropriateness for the female touch compared to the male one. Shaded areas represent 95% confidence intervals.

A summary of the results for Experiment 1 is reported in Table 3. Model selection indicated that the best-fitting model for Appropriateness included a three-way interaction between Avatar Gender, Avatar Ethnicity, and Body Area, Attractiveness and Trustworthiness ratings as covariates, and an Avatar Gender × RWA interaction term. Random effects allowed intercepts and slopes for Avatar Gender, Avatar Ethnicity, and Body Area to vary by participant (*R*^*2*^_*marginal*_ = 0.1, *R*^*2*^_*conditional*_ = 0.47). The model revealed an interaction between Avatar Gender and Area (χ^2^(2) = 31.85, *p* < .001). When touched by a female avatar, the intimate (*b* = −12.28, *SE* = 2.07, *t* = −5.92, *p* < .001, *d* = -0.66) and the neutral (*b* = −11.49, *SE* = 1.81, *t* = −6.34, *p* < .001, *d* = -0.62) areas were rated as less appropriate than the social area, but no difference between the neutral and intimate was found. When touched by a male avatar, the intimate area was rated as less appropriate than the neutral (*b* = −10.30, *SE* = 1.52, *t* = −6.77, p < .001, *d* = −0.55) and social (*b* = −12.74, *SE* = 2.07, *t* = −6.14, *p* < .001, *d* = -0.68), but no difference between neutral and social was found. The interaction between Avatar Gender and Avatar Ethnicity was also significant (χ^2^(1) = 11.19, *p* < .001). Post-hoc analyses showed a trend whereby touch from African females was perceived as less appropriate than from Caucasian females (*b* = −3.44, *SE* = 1.74, *t* = −1.98, *p* = .051, *d* = −0.18). A significant interaction between Avatar Gender and Right Wing Authoritarianism (RWA) was found (χ^2^(2) = 16.24, p < .001). Specifically, higher RWA scores predicted increased perceived appropriateness of touch for female avatars (β = 4.84, *SE* = 1.35, *CI* [2.16, 7.52]), but not for male avatars (β = 0.86, *SE* = 1.86, *CI* [−2.87, 4.60]; Fig. 3).

**Table 3:** Summary of the main results for Experiment 1 (Heterosexual Caucasian Men).

Outcome	Effect	Type	Statistic	p	Effect Size	Direction
**Appropriateness of touch**	**Avatar Gender × Area**	Interaction	χ^2^(2) = 31.85	< 0.001	—	—
	Female: Intimate vs. Social	Post-hoc	*t* = −5.92,	< 0.001	*d* = −0.66	Intimate < Social
			*b* = −12.28,			
			*SE* = 2.07			
	Female: Neutral vs. Social	Post-hoc	*t* = −6.34,	< 0.001	*d* = −0.62	Neutral < Social
			*b* = −11.49,			
			*SE* = 1.81			
	Male: Intimate vs. Neutral	Post-hoc	*t* = −6.77,	< 0.001	*d* = −0.55	Intimate < Neutral
			*b* = −10.30,			
			*SE* = 1.52			
	Male: Intimate vs. Social	Post-hoc	*t* = −6.14,	< 0.001	*d* = −0.68	Intimate < Social
			*b* = −12.74,			
			*SE* = 2.07			
	**Avatar Gender × Avatar Ethnicity**	Interaction	χ^2^(1) = 11.19	< 0.001	—	—
	African vs. Caucasian female	Post-hoc	*t* = −1.98,	= 0.051	*d* = −0.18	African < Caucasian (trend)
			*b* = −3.44,			
			*SE* = 1.74			
	**Avatar Gender × RWA**	Moderation	χ^2^(2) = 16.24	< 0.001	—	—
	RWA (female avatar)	Moderation	β = 4.84, *SE* = 1.35	< 0.001	95% CI [2.16, 7.52]	Higher RWA → more appropriate
**Pleasantness of touch**	**Attractiveness**	Covariate	χ^2^(1) = 15.20	< 0.001	β = 3.77, SE = 0.97	Higher attractiveness → greater pleasantness
	**Avatar Gender × Avatar Ethnicity × Area**	Interaction	χ^2^(2) = 8.20	= 0.017	—	—
	Intimate: Caucasian female vs. Caucasian male	Post-hoc	*t* = 2.11,	= 0.038	*d* = 0.57	Female > Male
			*b* = 9.15,			
			*SE* = 4.33			
	Intimate: African male vs. Caucasian male	Post-hoc	*t* = 2.71,	= 0.007	*d* = 0.36	African > Caucasian
			*b* = 5.76,			
			*SE* = 2.13			
**Disgust of touch**	**Attractiveness**	Covariate	χ^2^(1) = 10.98	< 0.001	β = −3.57, SE = 1.08	Higher attractiveness → lower disgust
	**Avatar Gender × Area**	Interaction	χ^2^(2) = 35.38	< 0.001	—	—
	Intimate: Female vs. Male avatar	Post-hoc	*t* = −2.11,	= 0.040	*d* = −0.49	Female < Male (less disgust)
			*b* = -9.60,			
			*SE* = 4.54			
**Erogeneity of touch**	**Attractiveness**	Covariate	χ^2^(1) = 6.58	= 0.010	β = 3.08, SE = 1.20	Higher attractiveness → higher erogeneity
	**Trustworthiness**	Covariate	χ^2^(1) = 18.31	< 0.001	β = 5.30, SE = 1.24	Higher trustworthiness → higher erogeneity
	**Avatar Gender × Avatar Ethnicity × Area**	Interaction	χ^2^(2) = 26.42	< 0.001	—	—
	Intimate: Caucasian female vs. Caucasian male	Post-hoc	t = 2.46, *b* = 15.17, *SE* = 6.16	= 0.016	*d* = 0.79	Female > Male
**SCR (log)**	**STQ**	Covariate	χ^2^(1) = 3.97	= 0.046	β = 0.27, SE = 0.14	Higher STQ → stronger SCR
	**Avatar Gender × Avatar Ethnicity × Area**	Interaction	χ^2^(2) = 20.31	< 0.001	—	—
	Intimate: African male vs. Caucasian male	Post-hoc	t = 3.36, *b* = 0.37, *SE* = 0.11	< 0.001	*d* = 0.43	African > Caucasian
	Social: African male vs. Caucasian male	Post-hoc	t = 3.51, *b* = 0.38, *SE* = 0.11	< 0.001	*d* = 0.45	African > Caucasian
	**Avatar Gender × Avatar Ethnicity × SDO**	Moderation	χ^2^(4) = 12.61	= 0.013	—	—
	Caucasian male vs. Caucasian female touch (high SDO)	Moderation	*t* = 2.54, *b* = 0.19, *SE* = 0.07	= 0.010	*d* = 0.22	Higher SDO → Male > Female (higher SCR)
**Heart Rate**	**Avatar Gender**	Main effect	χ^2^(1) = 6.02, *t* = −2.38, *b* = −0.66, *SE* = 0.28	= 0.014	*d* = −0.17	Female → stronger HR deceleration
	**Attractiveness**	Covariate	χ^2^(1) = 5.86	= 0.015	β = 0.33, SE = 0.13	Higher attractiveness → lower HR deceleration
**Interpersonal Distance**	**Avatar Gender × Avatar Ethnicity × Intergroup Disgust Sensitivity**	Moderation	χ^2^(4) = 20.64	<0.001	—	—
	African female vs. Caucasian female (high Intergroup Disgust Sensitivity)	Moderation	*t* = 2.20, *b* = 9.43, *SE* = 4.45	= 0.036	*d* = 0.44	Higher Intergroup Disgust Sensitivity → African Female > Caucasian Female (larger Distance)

*Note.* Effect sizes are reported as standardized regression coefficients (β) for covariates and Cohen's d for post-hoc comparisons. p-values for post-hoc tests are Tukey-corrected.

##### 3.1.1.2 Pleasantness of touch

Model selection indicated that the best-fitting model for Pleasantness included a three-way interaction between Avatar Gender, Avatar Ethnicity, and Body Area, and Attractiveness and Trustworthiness ratings as covariates. Random effects allowed intercepts and slopes for Avatar Gender and Avatar Ethnicity to vary by participant (*R*^*2*^_*marginal*_ = 0.08, *R*^*2*^_*conditional*_ = 0.54). The model revealed a significant main effect of Attractiveness (χ^2^(1) = 15.20, *p* < .001), indicating that higher levels of attractiveness were associated with greater touch pleasantness (β = 3.77, *SE* = 0.97, *t* = 3.90). This confirms that participants considered the touch of more attractive avatars as more pleasant, regardless of their gender, ethnicity, or the area of touch. Additionally, a significant three-way interaction was found between Avatar Gender, Avatar Ethnicity, and Area (χ^2^(2) = 8.20, *p* = .017). Pairwise comparisons revealed a significant effect in the intimate area: for Caucasian avatars, touch from female avatars was rated as significantly more pleasant than touch from male avatars (*b* = 9.15, *SE* = 4.33, *t* = 2.11, *p* = .038, *d* = 0.57); African male avatars were rated as significantly more pleasant than Caucasian male avatars (*b* = 5.76, SE = 2.13, *t* = 2.71, *p* = .007, *d* = 0.36). No other significant effects were found for the remaining conditions, including the neutral and social areas.

##### 3.1.1.3 Disgust of touch

Results for disgust ratings are reported in full in the Supplementary Materials; a summary is also provided in Table 3.

##### 3.1.1.4 Erogeneity of touch

**Fig. 4:**
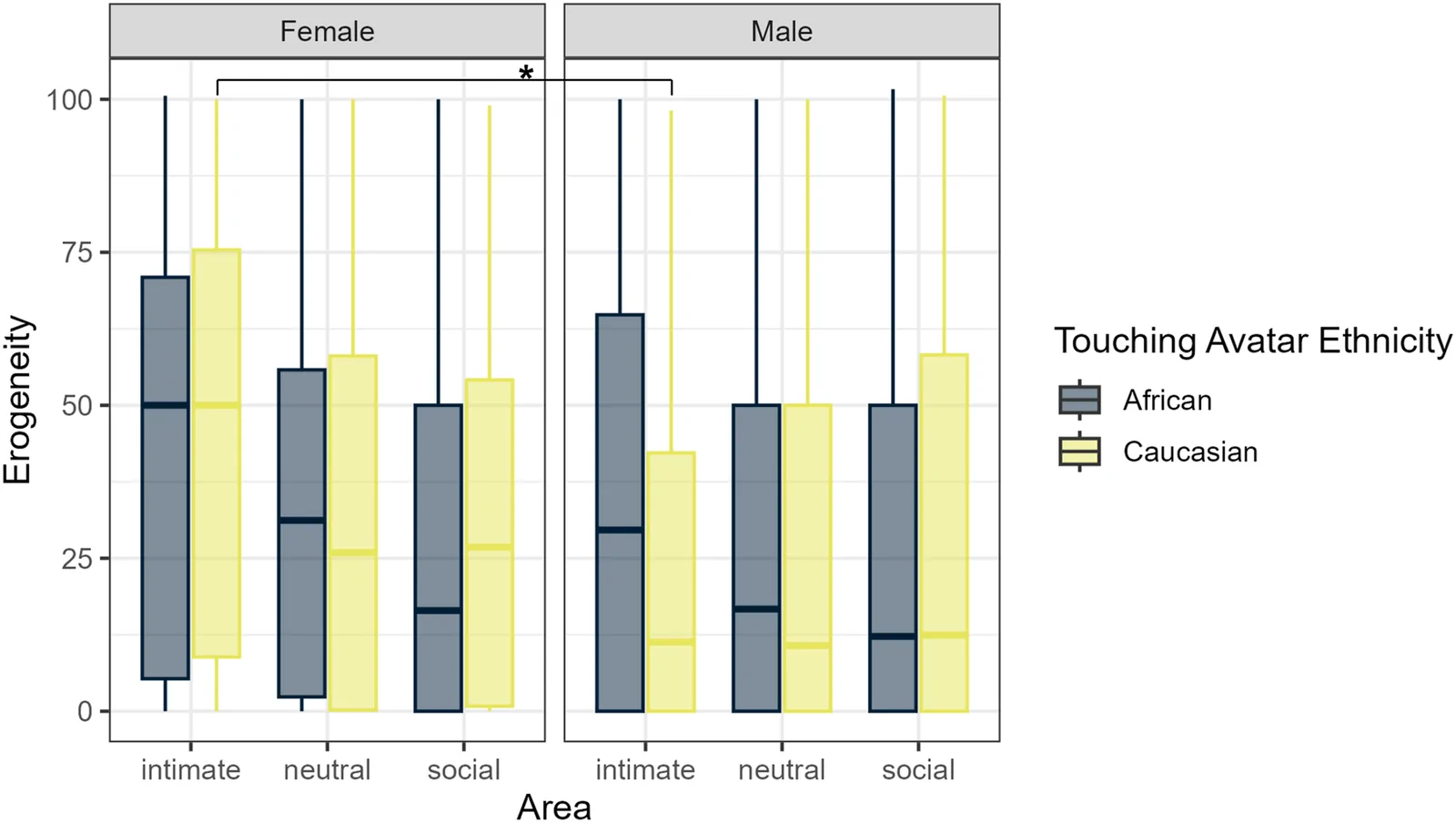
Erogeneity ratings in Heterosexual Caucasian Men. Boxplots display the median (horizontal line), interquartile range (IQR; box), 1.5× IQR (whiskers), and outliers (individual points). Asterisks indicate significant post-hoc differences for the Intimate area, in which participants rated the Caucasian female touch as more erogenous than the Caucasian male touch.

Model selection indicated that the best-fitting model for Erogeneity included a three-way interaction between Avatar Gender, Avatar Ethnicity, and Area, Attractiveness and Trustworthiness ratings as covariates, and an Avatar Gender × SDO interaction term. Random effects allowed intercepts and slopes for Avatar Gender and Avatar Ethnicity to vary by participant (*R*^*2*^_*marginal*_ = 0.094, *R*^*2*^_*conditional*_ = 0.64). The model revealed main effects of Attractiveness (χ^2^(1) = 6.58, *p* = .010) and Trustworthiness (χ^2^(1) = 18.31, *p* < .001), indicating that higher attractiveness (β = 3.08, *SE* = 1.20, *t* = 2.56) and trustworthiness (β = 5.30, *SE* = 1.24, *t* = 4.28) significantly predicted higher erogeneity ratings. A significant three-way interaction was also found between Avatar Gender, Avatar Ethnicity, and Area (χ^2^(2) = 26.42, *p* < .001). Post-hoc pairwise comparisons revealed that in the intimate touch area, participants rated touches from Caucasian female avatars as significantly more erogenous than those from Caucasian male avatars (*b* = 15.17, *SE* = 6.16, *t* = 2.46, *p* = .016, *d* = 0.79, Fig. 4). This suggests a stronger erogeneity response to intimate touch when the avatar is perceived as more similar to the participant in terms of ethnicity. Tellingly, no significant gender differences were observed for neutral (*p* = .815) or social (*p* = .366) touch areas within the Caucasian group, nor in any area for African avatars (all *p* > .05), indicating that the gender effect on erogeneity is primarily driven by ingroup perceptions.

#### 3.1.2 Physiological reactivity to touch

Because the residuals of the model were not normally distributed, a logarithmic transformation of the SCRs data was applied. After the transformation, the residuals met the assumption of normality. Model selection indicated that the best-fitting model for SCRs included a three-way interaction between Avatar Gender, Avatar Ethnicity, and Area, the STQ score as a covariate, and a three-way interaction between Avatar Gender, Avatar Ethnicity, and SDO. Random effects allowed intercepts and slopes for Avatar Gender and Avatar Ethnicity to vary by participant (*R*^*2*^_*marginal*_ = 0.048, *R*^*2*^_*conditional*_ = 0.61).

The model revealed a significant main effect of STQ (χ^2^(1) = 3.97, *p* = .046), indicating that higher social touch avoidance was associated with a stronger SCR (β = 0.27, SE = 0.14, *t* = 1.99). In other words, participants with higher STQ scores exhibited generally greater physiological arousal to the touch stimuli, regardless of the avatar's characteristics.

**Fig. 5:**
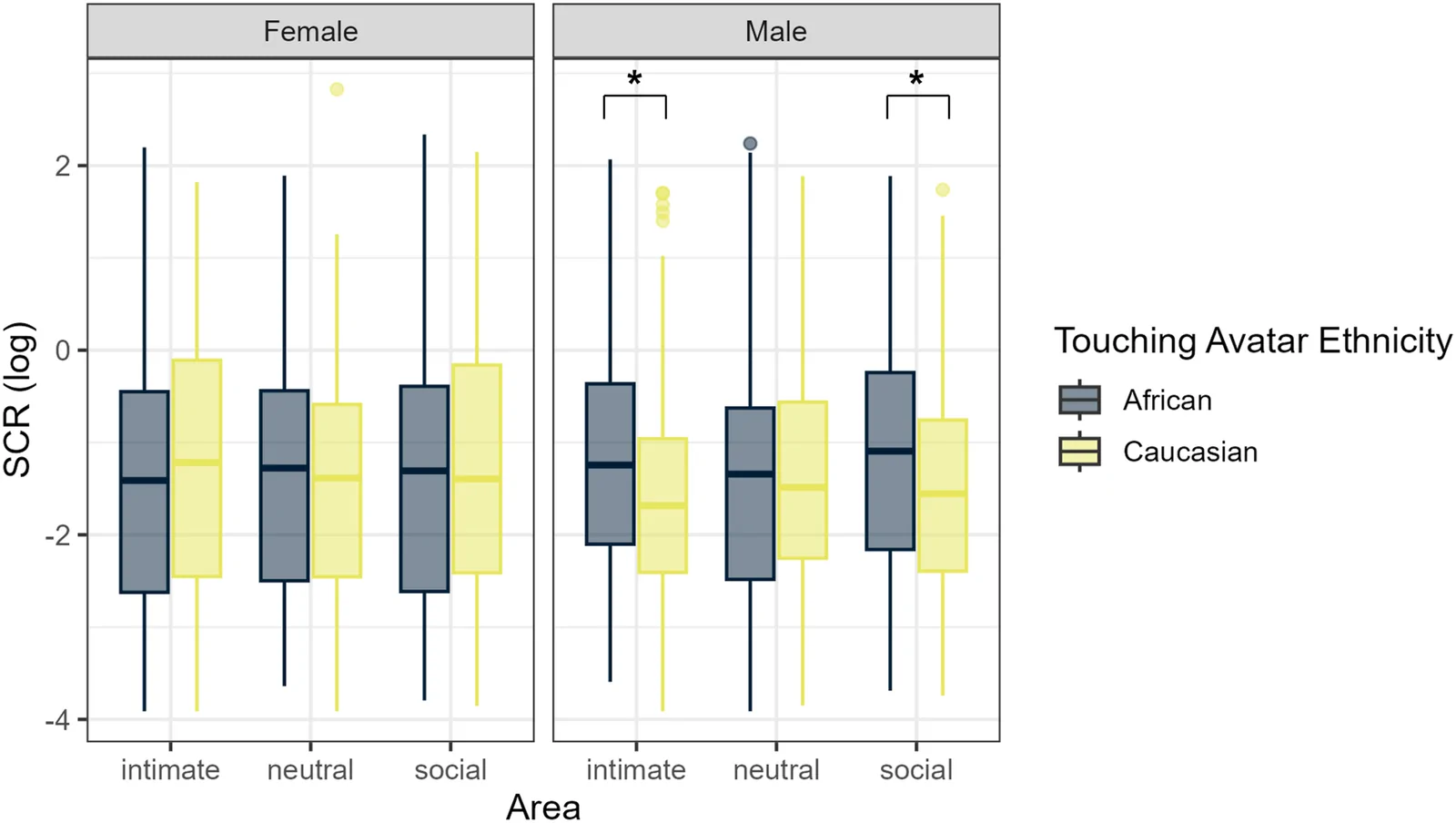
Skin conductance response (SCR, log-transformed) in Heterosexual Caucasian Men. Boxplots display the median (horizontal line), interquartile range (IQR; box), 1.5× IQR (whiskers), and outliers (individual points). Asterisks indicate significant post-hoc differences for intimate and social areas, with African male touch eliciting greater SCR than Caucasian male touch.

Additionally, a significant three-way interaction was observed between Avatar Gender, Avatar Ethnicity, and Area (χ^2^(2) = 20.31, *p* < .001), indicating that SCR varied based on the combination of these factors. Post-hoc analyses revealed that African male avatars elicited significantly higher SCR than Caucasian male avatars when touching intimate (*b* = 0.37, *SE=*0.11, *t* = 3.36, *p* < .001, *d* = 0.43) and social (*b* = 0.38, *SE* = 0.11, *t* = 3.51, *p* < .001, *d* = 0.45) areas (Fig. 5). This pattern suggests a heightened physiological response to avatars of different ethnicities, particularly in contexts involving intimate or social touch. A significant interaction between SDO, Avatar Gender, and Avatar Ethnicity was also observed (χ^2^(4) = 12.61, *p* = .013). This interaction reflected an increased SCR to ingroup male touch, compared to ingroup female touch, at higher levels of SDO (β = 0.19, *SE* = 0.07, *t* = 2.54, *p* = .01, *d* = 0.22), possibly reflecting a stronger sympathetic activation for the same-sex touch, which is generally perceived as less desirable among heterosexual men.

Concerning heart rate, model selection indicated that the best-fitting model included a three-way interaction between Avatar Gender, Avatar Ethnicity, and Area, and Attractiveness ratings as a covariate. Random effects allowed intercepts and slopes for Avatar Gender and Avatar Ethnicity to vary by participant (*R*^*2*^_*marginal*_ = 0.01, *R*^*2*^_*conditional*_ = 0.11). The model showed a significant main effect of Avatar Gender (χ^2^(1) = 6.02, *p* = .014), accounted for a stronger HR deceleration following female touch compared to the male touch (*b* = −0.66, *SE* = 0.28, *t* = −2.38, *p* = .019, *d* = −0.17). A significant effect of Attractiveness was also found (χ^2^(1) = 5.86, *p* = .015), with lower Attractiveness predicting stronger HR deceleration (β = 0.33, *SE* = 0.13, *t* = 2.42).

#### 3.1.3 Interpersonal distance

Model selection indicated that the best-fitting model for Interpersonal Distance included a three-way interaction between Avatar Gender, Avatar Ethnicity, and Intergroup Disgust, and the effect of Trustworthiness ratings as a covariate. Random effects allowed intercepts to vary by participant (*R*^*2*^_*marginal*_ = 0.31, *R*^*2*^_*conditional*_ = 0.73).

The model revealed a significant interaction between Avatar Gender, Avatar Ethnicity, and intergroup disgust sensitivity (χ^2^(4) = 20.64, p < .001). Specifically, for female avatars, the increase in Intergroup Disgust Sensitivity Scale score was associated with a significantly larger distance maintained for African avatars compared to Caucasian avatars (*b* = 9.43, *SE* = 4.45, *t* = 2.20, *p* = .036, *d* = 0.44).

##### 3.1.3.1 Correlation analyses

Appropriateness and pleasantness showed a high positive correlation (*r* = 0.62), suggesting that touches rated as more appropriate were also perceived as more pleasant (Table 4). Both appropriateness (*r* = −0.49) and pleasantness (*r* = −0.63) were negatively correlated with disgust, indicating that more appropriate and pleasant touches elicited less disgust. Erogeneity showed a high positive correlation with pleasantness and moderate positive correlations with appropriateness, while being moderately and negatively associated with disgust, reflecting that more erogenous touch locations were perceived as more pleasant but also less disgusting. IPD showed a moderate positive correlation with disgust and negative correlations with both appropriateness and pleasantness, suggesting that greater interpersonal distance was associated with less appropriate and less pleasant — yet more disgusting — touch perception. Physiological responses showed generally weak to very weak associations with the subjective ratings. SCR was very weakly and negatively correlated with appropriateness, and weakly and positively correlated with disgust and IPD, suggesting a modest autonomic response to less appropriate and more distancing touch contexts. HR showed weak negative correlations with appropriateness and very weak positive correlations with IPD and SCR, indicating a limited correspondence between cardiac activity and touch perception.

**Table 4:** Correlations between touch evaluation, physiological indices and interpersonal distance for Experiment 1 (Heterosexual Caucasian Men).

Variable	Appropriateness	Pleasantness	Disgust	Erogeneity	IPD	SCR
**Appropriateness**	—					
**Pleasantness**	0.62***	—				
**Disgust**	−0.49***	−0.63***	—			
**Erogeneity**	0.32***	0.70***	−0.34***	—		
**IPD**	−0.26***	−0.29***	0.41***	−0.10*	—	
**SCR**	−0.09*	−0.01	0.14**	0.01	0.18***	—
**HR**	−0.13**	−0.03	0.07	0.03	0.09*	0.12**

*Note*. *p < .05. **p < .01. ***p < .001. p-values are FDR-corrected (Benjamini-Hochberg).

##### 3.1.3.2 Discussion Experiment 1

In the sample of heterosexual Caucasian men, touch responses were found to be modulated by social characteristics of the avatars, particularly gender, ethnicity, and subjective ratings of attractiveness and trustworthiness. Experiment 1 provided partial support for our hypotheses. The prediction that touches from female avatars would be evaluated more positively was supported, whereas the prediction of a general preference for ingroup touch received only limited support and emerged mainly in specific combinations of toucher gender, body area, and individual differences.

Consistent with participants’ sexual orientation, female avatars were generally perceived as more attractive than male avatars, a preference that was reflected in affective responses to touch. Interestingly, evaluations like perceived attractiveness and trustworthiness further influenced affective touch judgments. Specifically, touch was judged to be more pleasant and less disgusting when the avatar was deemed aesthetically pleasing. The effect also seemed to extend to the erotic component of touch, which increased both in the presence of avatars considered attractive and when they were judged trustworthy.

The interpersonal distance from the avatars was modulated by trustworthiness. In fact, avatars judged to be more trustworthy were allowed to get closer to the participants. Another interesting result concerns the effect of intergroup disgust sensitivity: higher scores on this scale were found to be associated with greater physical distance toward African female avatars than Caucasian female ones. This could indicate that, in subjects more sensitive to contact with outgroup members, more pronounced defensive reactions emerge in situations simulating intimate bodily interaction. The absence of significant interpersonal distance modulations toward male avatars may reflect a ceiling effect, whereby male avatars—especially when perceived as less attractive or trustworthy—are already kept at maximally safe distances, leaving limited room for further variation due to individual traits or perceived trustworthiness.

A more complex picture emerges from physiological responses. In line with our prediction that outgroup touch could elicit stronger autonomic responses, touch by African male avatars, especially in intimate or social body areas, elicited greater skin conductance responses (SCRs). Yet, this increased physiological response is not accompanied by more negative subjective evaluations of touch, in terms of pleasantness or disgust. One interpretation is that such activation might suggest an alertness or attention response related to the novelty of the stimulus, rather than racial bias. Participants' lower familiarity with being touched by black men in intimate or social areas might have contributed to increased physiological activation, without necessarily implying a negative affective evaluation. In line with this interpretation, SCR has been associated with the involuntary orienting response, a neurophysiological mechanism that enhances processing of unexpected or unfamiliar stimuli (Zimmer & Richter, 2023). Alternatively, increased electrodermal activity could reflect a dissociation between implicit (physiological) and explicit responses (VAS). A previous study showed that Dutch participants exhibited greater skin conductance level and maintained a greater physical distance from African than from Caucasian avatars, while reporting no differences in explicit judgments of agreeableness (Dotsch & Wigboldus, 2008). In that study, the physiological response was moderated by the level of implicit bias, suggesting that autonomic activation may reveal underlying biases not detected by self-reported measures. Moreover, we found an increased HR deceleration for female touch, suggesting a stronger orienting response for opposite-sex contact, consistent with greater social salience attributed to female touch. In contrast, HR deceleration was also stronger for low-attractive avatars, which may reflect increased vigilance in response to less desirable stimuli.

It is also worth noting that participants' individual dispositions, measured by questionnaires, further clarified the variability of responses. For instance, high scores on the RWA were found to be related to higher ratings of appropriateness of touch by female avatars, but not by male avatars. This result suggests that individuals who have a greater propensity for deference to constituted authority may react more negatively to physical touch by other men. This may reflect an internalized hierarchical pattern in which the female gender is less threatening, while the male gender is perhaps viewed as a symbolic threat to one's social position, highlighting a pattern consistent with rigid traditional gender norms. Finally, scores on the Social Touch Questionnaire (STQ) were found to be associated with increased SCRs to touch, regardless of avatar characteristics. This result suggests that individual differences in how physical touch is experienced may amplify physiological responsiveness, even in virtual environments, reinforcing the idea that tactile interactions are influenced by stable personality traits. Overall, the results of Experiment 1 indicate that, in the male sample, responses to touch are not driven by rigid or unidirectional racial biases, but rather by a complex interplay of familiarity, gender-related preferences, psychological dispositions, and internalized cultural norms. Thus, Experiment 1 supported the hypotheses concerning more positive responses to female touch among Caucasian heterosexual men and physiological sensitivity to outgroup touch, while providing only partial support for ingroup touch preference and greater interpersonal distance from outgroup avatars.

## 4 Experiment 2: heterosexual caucasian women

### 4.1 Results

#### 4.1.1 Virtual touch task

##### 4.1.1.1 Appropriateness of touch

A summary of the results for Experiment 2 is reported in Table 5. Model selection indicated that the best-fitting model for Appropriateness included a three-way interaction between Avatar Gender, Avatar Ethnicity and Area, and Attractiveness ratings as covariate. Random effects allowed intercepts and slopes for Avatar Gender, Avatar Ethnicity, and Area to vary by participant (*R*^*2*^_*marginal*_ = 0.34, *R*^*2*^_*conditional*_ = 0.71). The model revealed a significant main effect of Attractiveness (χ^2^(1) = 11.91, p < .001), with a positive association between Attractiveness and Appropriateness (β = 2.20, *SE* = 0.64, *t =* 3.45, p < .001). This indicates that touch from avatars perceived as more attractive was generally rated as more appropriate. Additionally, a significant interaction was found between Avatar Gender and Area (χ^2^(2) = 8.05, *p* = .018). Pairwise comparisons within each area revealed that touch from female avatars was rated as more appropriate than from male avatars in the neutral (*b* = 4.84, SE=1.55, *t* = 3.13, *p* = .002, *d* = 0.29) and social (*b* = 7.20, SE = 1.55, *t* = 4.66, *p* < .0001, *d* = 0.43) areas, while no significant difference was found in the intimate area (*b* = 2.39, SE = 1.55, *t* = 1.54, *p* = .125, *d* = 0.14).

**Table 5:** Results summary for Experiment 2 (Heterosexual Caucasian Women).

Outcome	Effect	Type	Statistic	p	Effect Size	Direction
**Appropriateness**	**Attractiveness**	Covariate	χ^2^(1) = 11.91	< 0.001	β = 2.20, *SE* = 0.64	Higher attractiveness → more appropriate
	**Avatar Gender × Area**	Interaction	χ^2^(2) = 8.05	= 0.018	—	—
	Neutral: Female vs. Male avatar	Post-hoc	*t* = 3.13, *b* = 4.84, *SE* = 1.55	= 0.002	*d* = 0.29	Female > Male
	Social: Female vs. Male avatar	Post-hoc	*t* = 4.66, *b* = 7.20, *SE* = 1.55	< 0.001	*d* = 0.43	Female > Male
**Pleasantness**	**Attractiveness**	Covariate	χ^2^(1) = 77.23	< 0.001	β = 5.30, *SE* = 0.60	Higher attractiveness → greater pleasantness
	**Avatar Gender × Ethnicity**	Interaction	χ^2^(1) = 19.59	<0.001		
	African vs. Caucasian female avatar	Post-hoc	*t* = 3.54, *b* = 3.89, SE = 1.10	< 0.001	*d* = 0.24	African > Caucasian
	**IAT → Attractiveness → Pleasantness (ACME)**	Mediation	*b* = 0.22, 95% CI [0.05, 0.43]	= 0.005	67% mediated	Full mediation via attractiveness
**Disgust**	**Attractiveness**	Covariate	χ^2^(1) = 13.60	< 0.001	β = −2.91, *SE* = 0.79	Lower attractiveness → more disgusting
	**Avatar Gender × Ethnicity**	Interaction	χ^2^(1) = 6.16	= 0.013	—	—
	African vs. Caucasian female avatar	Post-hoc	*t* = −2.17, *b* = −3.22, *SE* = 1.48	= 0.032	*d* = −0.16	African < Caucasian
	**Avatar Gender × Ethnicity × Area**	Interaction	χ^2^(2) = 6.22	= 0.045	—	—
	Neutral: African vs. Caucasian female avatar	Post-hoc	*t* = -2.51, *b* = −5.58, *SE* = 2.22	= 0.012	*d* = −0.28	African < Caucasian
**Erogeneity**	**Avatar Gender**	Main effect	χ^2^(1) = 8.11, *t* = 2.85, *b* = 3.75, *SE* = 1.32	= 0.004	*d* = 0.23	Male > Female
	**Attractiveness**	Covariate	χ^2^(1) = 76.17	< 0.001	β = 5.81, *SE* = 0.66	Higher attractiveness → more erogenous
**SCR (log)**	**STQ**	Covariate	χ^2^(1) = 5.09, *t* = −2.25	= 0.024	β = −0.23, *SE* = 0.12	Lower touch avoidance → stronger SCR
	**Avatar Gender × Ethnicity × Area**	Interaction	χ^2^(2) = 14.28	< 0.001	—	—
	Neutral: African vs. Caucasian female avatar	Post-hoc	*t* = 2.10, *b* = 0.21, *SE* = 0.09	= 0.037	*d* = 0.26	African > Caucasian
**Heart Rate**	**Area**	Main effect	χ^2^(2) = 11.53	= 0.003	—	—
	Intimate vs. Neutral	Post-hoc	*t* = −3.31, *b* = −0.65, *SE* = 0.20	= 0.003	*d* = −0.18	Intimate → greater HR deceleration
	**SDO × Avatar Ethnicity**	Moderation	χ^2^(2) = 8.73	= 0.013	—	—
	Higher SDO: ingroup vs. outgroup touch	Moderation	*t* = 2.32, *b* = 0.43, *SE* = —	= 0.020	*d* = 0.12	Ingroup → greater HR deceleration
**Interpersonal Distance**	**Avatar Gender**	Main effect	χ^2^(1) = 7.38	= 0.007	—	—
	Female vs. Male	Post-hoc	*t* = −2.71, *b* = −6.82, *SE* = 2.52	= 0.008	*d = −0.42*	Female < Male
	**Attractiveness**	Covariate	χ^2^(1) = 7.00	= 0.008	β = −4.98, *SE* = 1.88	Higher attractiveness → shorter distance
	**Trustworthiness**	Covariate	χ^2^(1) = 9.09	= 0.003	β = −7.13, *SE* = 2.36	Higher trustworthiness → shorter distance

*Note.* Effect sizes are reported as standardized regression coefficients (β) for covariates and Cohen's d for post-hoc comparisons. p-values for post-hoc tests are Tukey-corrected.

##### 4.1.1.2 Pleasantness of touch

**Fig. 6:**
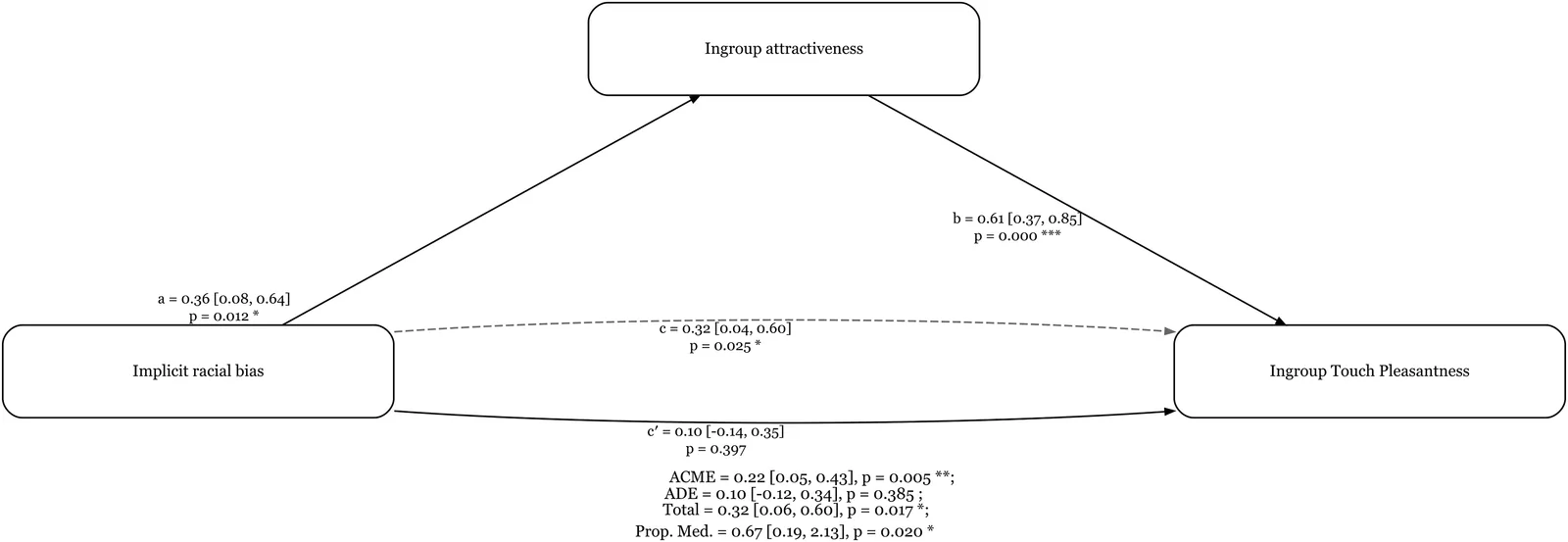
Caucasian heterosexual women (Experiment 2). Standardized mediation model. Higher D scores in the IAT predicted greater attractiveness for the ingroup male avatar compared to the outgroup one (path a), which in turn predicted greater ingroup male touch pleasantness compared to the outgroup one (path b). The indirect effect (a·b; ACME) was significant, while the direct effect of IAT on pleasantness (c′) was not; the total effect (c) remained positive. Values show estimates with 95% CIs and p-values.

Model selection indicated that the best-fitting model for Pleasantness included a three-way interaction between Avatar Gender, Avatar Ethnicity and Area, and Attractiveness ratings as covariate. Random effects allowed intercepts and slopes for Avatar Gender, Avatar Ethnicity, and Area to vary by participant (*R*^*2*^_*marginal*_ = 0.19, *R*^*2*^_*conditional*_ = 0.63). The model revealed a significant main effect of Attractiveness (χ^2^(1) = 77.23, p < .001), indicating that touch from avatars perceived as more attractive were generally rated as more pleasant (β = 5.30, SE = 0.60, *t* = 8.79, *p* < .001). A significant interaction between Avatar Gender and Avatar Ethnicity (χ^2^(1) = 19.59, *p* < .001) emerged, with pairwise comparisons revealing that touches from African female avatars were rated as more pleasant than touches from Caucasian female avatars (*b* = 3.89, *SE* = 1.10, *t* = 3.54, *p* < .001, *d* = 0.24), while the difference was not significant for male avatars (*b* = −2.19, *SE* = 1.14, *t* = −1.92, *p* = .057, *d* = −0.14). Finally, a main effect of Area was observed (χ^2^(2) = 74.91, *p* < .001), indicating that interactions in intimate areas were rated as less pleasant compared to neutral (*b* = −11.5, SE = 2.24, *t* = −5.14, *p* < .001, *d* = −0.72) and social areas (*b* = −24.7, *SE* = 2.93, *t* = −8.44, *p* < .001, *d* = −1.56), that in turn were rated as significantly more pleasant than neutral ones (*b* = 13.20, *SE* = 1.91, *p* < .001, *d* = 0.83). A mediation analysis was conducted to investigate the relationship between implicit bias, perceived attractiveness, and pleasantness ratings. The IAT score was used as a measure of implicit ingroup preference, while an attractiveness index was computed to reflect the difference in perceived attractiveness between Caucasian and African avatars for each participant. This index captures the relative preference for avatars of the same ethnic group and was calculated by subtracting the average attractiveness ratings of African male avatars from those of Caucasian male avatars, resulting in a positive score for participants who perceived ingroup avatars as more attractive. The analysis revealed a significant indirect effect of the IAT on pleasantness ratings, suggesting that implicit bias in favor of the ingroup influenced the perception of avatar attractiveness, which in turn affected pleasantness evaluations (ACME: β = 0.22, 95% CI [0.05, 0.43], *p* = .005, see Fig. 6). In contrast, the direct effect of the IAT on pleasantness, after accounting for the attractiveness mediator, was not significant (ADE: β = 0.10, 95% CI [−0.12, 0.34], *p* = .385), indicating that the effect was fully mediated by perceived attractiveness. Notably, 67% of the total effect of IAT on pleasantness was mediated through attractiveness (95% CI [19%, 213%], *p* = .020), emphasizing the central role of attractiveness in shaping pleasantness ratings.

##### 4.1.1.3 Disgust of touch

Results for disgust ratings are reported in full in the Supplementary Materials; a summary is also provided in Table 5.

##### 4.1.1.4 Erogeneity of touch

Model selection indicated that the best-fitting model for Erogeneity included a three-way interaction between Avatar Gender, Avatar Ethnicity and Area, and Attractiveness ratings as covariate. Random effects allowed intercepts and slopes for Avatar Gender, Avatar Ethnicity, and Area to vary by participant. The model (*R*^*2*^_*marginal*_ = 0.1, *R*^*2*^_*conditional*_ = 0.61) revealed significant main effects of Avatar Gender (χ^2^(1) = 8.11, *p* = .004), Area (χ^2^(2) = 19.15, *p* < .001), and Attractiveness (χ^2^(1) = 76.17, p < .001). Regarding Avatar Gender, touches from male avatars were rated as significantly more erogenous than touches from female avatars (*b* = 3.75, *SE* = 1.32, *p* = .006, *d* = 0.23). For the main effect of Area, intimate touches were rated as significantly more erogenous than both neutral (*b* = 11.18, *SE* = 2.70, *t* = 4.14, *p* < .001, *d =* 0.68) and social touches (*b* = 13.95, *SE* = 3.21, *t* = 4.34, *p* = .001, *d =* 0.85), while no significant difference was found between neutral and social touches (*b* = 2.78, *SE* = 1.41, p = .132, *d =* 0.17). Finally, the strong main effect of Attractiveness indicates that touches from more attractive avatars were rated as substantially more erogenous (β = 5.81, *SE* = 0.66, *t* = 8.73).

#### 4.1.2 Physiological reactivity to touch

Model selection indicated that the best-fitting model for SCRs included a three-way interaction between Avatar Gender, Avatar Ethnicity and Area, and STQ as covariate. Random effects allowed intercepts and slopes for Avatar Gender and Avatar Ethnicity to vary by participant. The model (*R*^*2*^_*marginal*_ = 0.005, *R*^*2*^_*conditional*_ = 0.53) showed a main effect of STQ (χ^2^(1) = 5.09, *p* = .024), such that lower social touch avoidance was associated with stronger SCRs (*b* = −0.24, *SE* = 0.11, *t* = −2.25). In contrast to men, lower touch avoidance in women may reflect greater attentional engagement with touch, yielding larger SCRs. We also observed a significant three-way interaction between Avatar Gender, Avatar Ethnicity, and Area (χ^2^(2) = 14.28, *p* < .001). Post-hoc analyses revealed a significant difference in the neutral touch condition for female avatars, with African avatars eliciting a significantly higher SCR compared to Caucasian avatars (*b* = 0.21, *SE* = 0.09, *t* = 2.10, *p* = .037). No other significant effects were found.

**Fig. 7:**
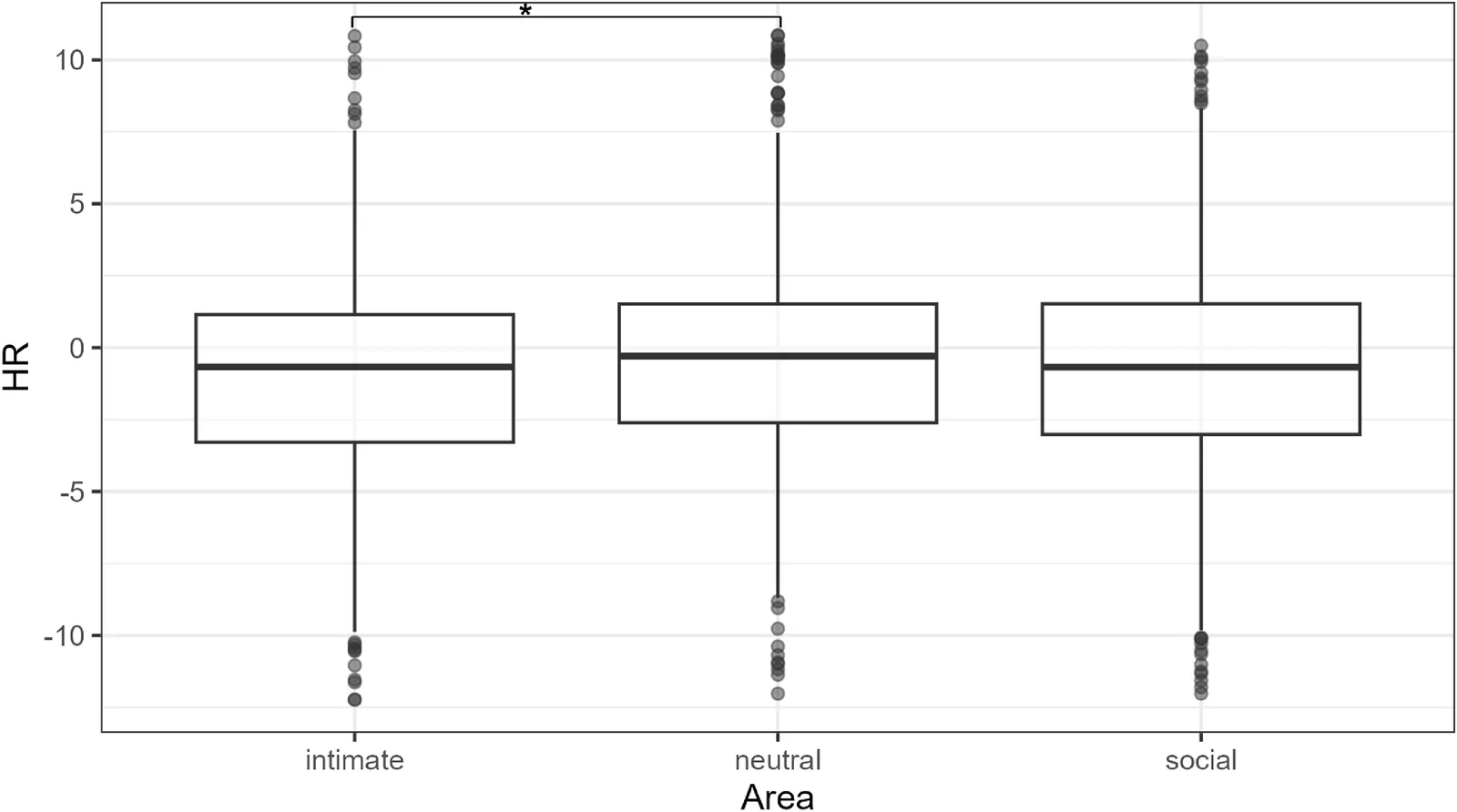
Caucasian heterosexual women (Experiment 2). Heart rate responses as a function of touch area (intimate, social, neutral). Boxplots display the median (horizontal line), interquartile range (IQR; box), 1.5× IQR (whiskers), and outliers (individual points). Touches applied to intimate areas elicited significantly greater heart rate deceleration compared to neutral areas, while differences between intimate and social areas and between social and neutral areas did not reach significance.

**Fig. 8:**
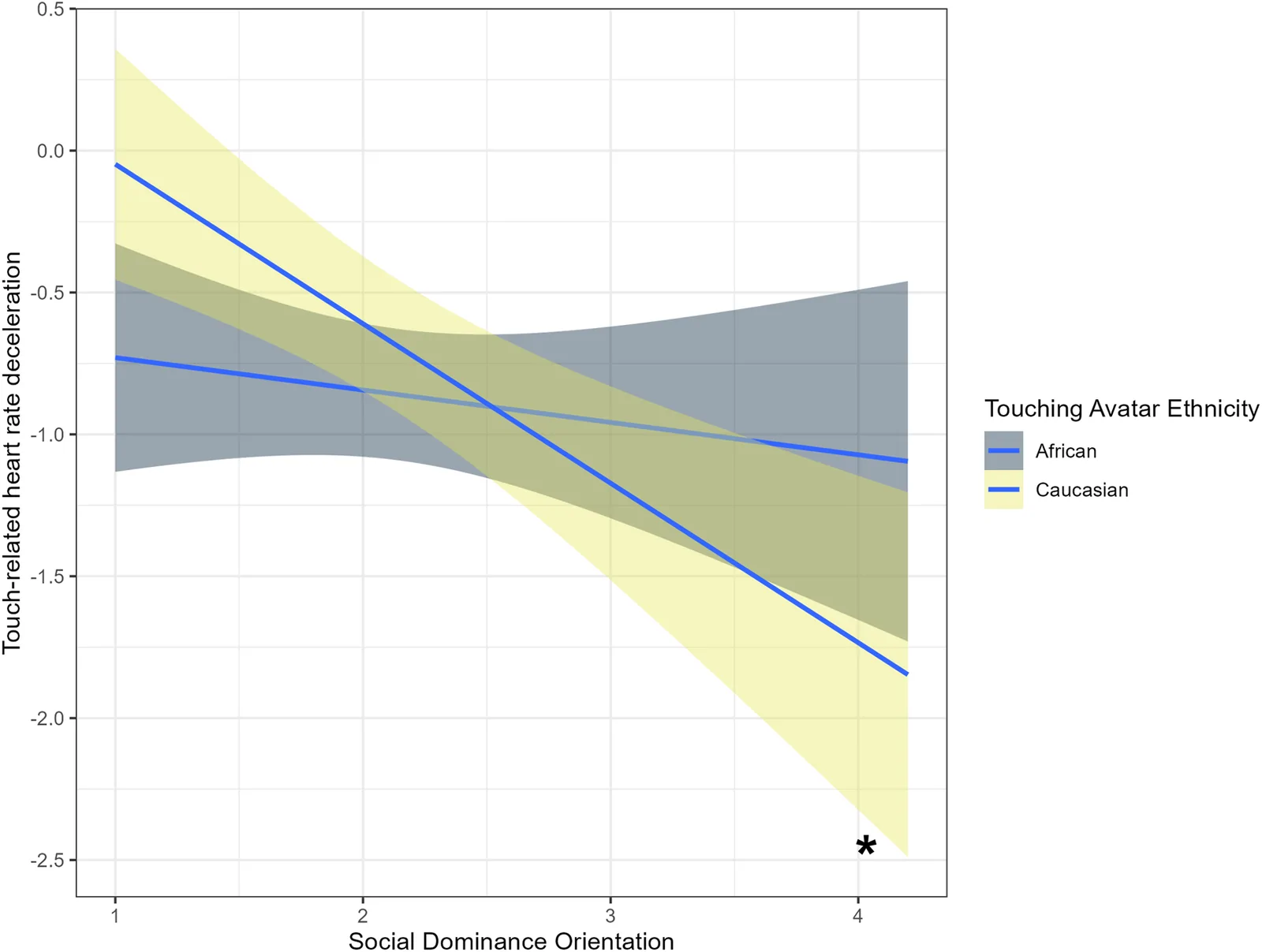
Caucasian heterosexual women (Experiment 2). Higher levels of social dominance orientation-reflecting a stronger preference for group-based hierarchies and inequality - were associated with increased heart rate deceleration for the ingroup touch compared to the outgroup one. Shaded areas represent 95% confidence intervals.

Model selection indicated that the best-fitting model for HR included a three-way interaction between Avatar Gender, Avatar Ethnicity and Area, and the interaction between Avatar Ethnicity and SDO. Random effects allowed intercepts and slopes for Avatar Gender to vary by participant. The model (*R*^*2*^_*marginal*_ = 0.014, *R*^*2*^_*conditional*_ = 0.088) revealed a main effect of Area (χ^2^(2) = 11.53, *p* = .003), accounted for a greater heart rate deceleration for touch in the intimate areas compared to the neutral areas (*b* = −0.65, *SE* = 0.20, *t* = −3.31, *p* = .003, *d* = −0.18, see Fig. 7), while the differences between intimate and social areas (*b* = −0.22, *SE* = 0.20, *t* = −1.08, *p* = .521, *d* = −0.06) and between neutral and social areas (*b* = 0.44, *SE* = 0.20, *t* = 2.20, *p* = .069, *d* = 0.11) were not significant. Additionally, a significant interaction between SDO and Avatar Ethnicity was found (χ^2^(2) = 8.73, *p* = .013). Trend analyses showed that higher SDO levels were associated with increased HR deceleration for the ingroup touch compared to the outgroup one (*b* = 0.43, *t* = 2.32, *p* = .02, *d* = 0.12, see Fig. 8).

#### 4.1.3 Interpersonal distance

Model comparison indicated that the best-fitting model for IPD included the two-way interaction between Avatar Gender and Avatar Ethnicity, Attractiveness and Trustworthiness as covariates, and the interaction between Avatar Gender and SDO. Random effects allowed intercepts to vary by participant. The model (*R*^*2*^_*marginal*_ = 0.09, *R*^*2*^_*conditional*_ = 0.84) for IPD in the women sample revealed significant main effects of Avatar Gender (χ^2^(1) = 7.38, *p* = .007), Attractiveness (χ^2^(1) = 7.00, *p* = .008), and Trustworthiness (χ^2^(1) = 9.09, *p* = .003). Specifically, participants maintained shorter distances from female compared to male avatars (*b* = −6.82, *SE* = 2.52, *t* = −2.71, *p* = .008), suggesting a general preference for proximity to female avatars. Additionally, higher attractiveness and trustworthiness scores were associated with shorter distances (Attractiveness: β = −4.98, SE = 1.88, *t* = −2.65; Trustworthiness: β = −7.13, SE = 2.36, *t* = −3.02), indicating that participants were more comfortable approaching avatars perceived as both attractive and trustworthy.

##### 4.1.3.1 Correlation analyses

Among women, appropriateness and pleasantness showed a high positive correlation (*r* = 0.71, Table 6), and both were highly and negatively associated with disgust (*r* = −0.56; *r* = −0.72), replicating the pattern observed in men. Erogeneity showed only a weak positive correlation with pleasantness (*r* = 0.20) and no significant associations with appropriateness (*r* = 0.02) or disgust (*r* = −0.005), suggesting that for women, the erogenous nature of the touched body region was largely unrelated to how appropriate or disgusting the touch was perceived. IPD showed a weak negative correlation with appropriateness (*r* = −0.17) and erogeneity (*r* = −0.17). and moderate correlation with pleasantness (*r* = −0.26) and disgust (*r* = 0.27), indicating that greater interpersonal distance was associated with less favorable and more aversive touch perception. Neither SCR nor HR showed any significant correlations with the subjective ratings or with IPD among women, suggesting that physiological arousal did not systematically co-vary with touch perception in this group.

**Table 6:** Correlations between touch evaluation, physiological indices and interpersonal distance for Experiment 2 (Heterosexual Caucasian Women).

Variable	Appropriateness	Pleasantness	Disgust	Erogeneity	IPD	SCR
**Appropriateness**	—					
**Pleasantness**	0.71***	—				
**Disgust**	−0.56***	−0.72***	—			
**Erogeneity**	0.02	0.20***	−0.00	—		
**IPD**	−0.17***	−0.26***	0.27***	−0.17***	—	
**SCR**	−0.08	0.02	−0.09	0.04	−0.00	—
**HR**	0.05	0.03	−0.04	−0.07	0.06	−0.07

*Note*. *p < .05. **p < .01. ***p < .001. p-values are FDR-corrected (Benjamini-Hochberg).

##### 4.1.3.2 Discussion Experiment 2

The results indicate that the perceived attractiveness of avatars plays a crucial role in modulating the experience of touch in virtual reality among heterosexual Caucasian women, significantly influencing its appropriateness, pleasantness, erogeneity, and disgust associated with the interaction. Experiment 2 provided partial support for our hypotheses. The prediction that female touch would be evaluated more positively was supported for appropriateness and disgust, especially in neutral and social areas, whereas the prediction of a generalized preference for ingroup touch was not supported. Touch from avatars judged to be more attractive was rated as more appropriate and more pleasant, but also as less disgusting and more erogenous, suggesting that social evaluation of the virtual agent profoundly affects the affective and bodily processing of touch. Notably, outgroup male avatars were rated as more attractive than ingroup male avatars. However, this effect was moderated by implicit racial bias: women with stronger implicit bias reversed this pattern, showing reduced attractiveness ratings for outgroup male avatars. Mediation analyses further revealed that implicit preferences for one's ethnic ingroup, as measured by the Implicit Association Test, did not directly predict the pleasantness of touch, but operated indirectly through avatar attractiveness. This supports our hypothesis that prejudice-related individual differences would modulate responses to outgroup touch, although the effect emerged through aesthetic evaluation rather than through a direct effect of touch pleasantness. These findings support the idea that perceived attractiveness and implicit biases are tightly intertwined (Rudman & McLean, 2016), and extend the observation that the perceived attractiveness of the caresser modulates the hedonic value of touch (Novembre et al., 2021). The effect of attractiveness also extends to the interpersonal distance maintained by the avatar, with greater closeness toward avatars perceived as more attractive and trustworthy. Interpersonal distance, in turn, predicts touch ratings, suggesting it may act as a precursor to social touch evaluation. However, the hypothesis that Caucasian women would maintain larger distance from outgroup avatars was not supported. Instead, interpersonal distance was primarily shaped by avatar gender and aesthetic and moral evaluations. Consistent with previous studies (Fusaro et al., 2021), touches from female avatars were considered more appropriate than male avatars and less disgusting, particularly in neutral and social areas. These findings are part of a large body of literature showing that the gender of the toucher represents a powerful top-down modulator of the meaning attributed to touch: female touch is indeed more frequent in everyday life and generally more accepted by both sexes (Stier & Hall, 1984; Suvilehto et al., 2015), and women show greater tolerance and familiarity with even intimate forms of physical touch (Roese et al., 1992). Consistently, participants indicated an implicit preference for close interaction with female avatars. When the dimension of erogeneity is considered, the pattern reverses: male avatars were perceived as significantly more erogenous than female ones, a finding consistent with the sample's heterosexual orientation, suggesting that sexual attraction may override more generalized social preferences. The body area touched is confirmed to be a powerful modulator of the affective experience of touch, significantly influencing its subjective perception and physiological response. In line with the existing literature (Suvilehto et al., 2023), which emphasizes the existence of implicit rules about which parts of the body are considered appropriate to touch depending on the context and the relationship between the people involved, our results show that touch in intimate areas is perceived qualitatively differently than touch in neutral or social areas. In particular, touch in intimate areas was judged significantly less pleasant and more disgusting, confirming a lower social and affective acceptability of touch in these areas, especially in the absence of a strong relational bond. However, the intimacy of the area touched was associated with greater erogeneity, suggesting a dissociation between the positive affective (pleasantness) and sexual components of touch.

Physiologically, touch in intimate areas produced a more pronounced deceleration of heart rate than touch in neutral areas. This pattern of autonomic response suggests deeper affective involvement and greater emotional relevance of touch in these areas of the body. Heart rate deceleration can reflect a reduction in cardiac activity associated with an attentional orientation toward emotionally salient stimuli (Bradley et al., 2001). Consistent with this interpretation, intimate touches localized on the chest and pelvic area appear to have elicited greater attention than neutral touches on the knee and foot, highlighting how the affective and social valence of the body area profoundly influences the physiological response to tactile interaction. Heart rate responses were also modulated by the interaction between touching avatar ethnicity and participants' social dominance orientation, such that women higher in social dominance orientation showed stronger heart rate deceleration during ingroup touch. This result may be interpreted as increased attention orienting to the ingroup touch, which is in line with studies in face recognition reporting preferential attention for ingroup versus outgroup faces (Kawakami et al., 2017). The role of the avatar's ethnicity modulates the experience of touch, revealing somewhat divergent responses between the explicit and implicit levels. In particular, touch exerted by African female avatars was found to be more pleasant and less disgusting than that performed by Caucasian female avatars, especially when performed on the neutral areas of the body. From a physiological point of view, neutral touch on the knee and foot by African women elicited increased SCR compared to the same touch performed by Caucasian women, suggesting greater autonomic activation in response to this type of interaction. This divergence may reflect a distinction between different types of processing: on the one hand, a conscious, positive evaluation of touch (pleasantness and reduced disgust); on the other, an amplified physiological response, potentially related to implicit factors such as novelty and salience of African avatars. Another explanation could be related to social desirability, whereby participants may have responded explicitly according to social norms in order not to appear judgmental toward another ethnic group, while at a more implicit and less controllable level they may have shown more distrust and activation when faced with black avatars. Overall, Experiment 2 supported the hypotheses concerning more positive evaluations of female touch, the role of prejudice-related individual differences, and physiological sensitivity to outgroup touch. However, it did not support a generalized ingroup preference in explicit touch evaluations and interpersonal distance. Instead, women's responses were primarily shaped by avatar attractiveness, gender, and body area, while ethnicity-related effects emerged in more specific forms through attractiveness-mediated implicit bias and heightened physiological responses to African female touch in neutral body areas.

## 5 General discussion

Intergroup bias is a pervasive process that affects attitudes, cognition, and behaviors towards people belonging to different social groups. Yet, the extent to which such bias and racial prejudice are embodied in tactile interactions with outgroups remains poorly understood. The present studies leveraged Immersive Virtual Reality to recreate controlled, ecologically valid tactile interactions with outgroup members. Heterosexual Caucasian men (experiment 1) and heterosexual Caucasian women (experiment 2) observed a gender-congruent virtual body aligned with their real one, being caressed in intimate, social, and neutral body parts by African and Caucasian male and female avatars. Across both experiments, responses to touch were shaped by a complex interplay of touching avatar gender, ethnicity, perceived attractiveness and trustworthiness, and individual differences in social attitudes of the participants. In both men and women, avatars rated as more attractive and trustworthy generally elicited more positive affective evaluations as well as reduced interpersonal distance, highlighting the central role of social evaluation in tactile interactions.

Heterosexual men participants showed heightened physiological responses to touch from African male avatars, and greater distancing from African female avatars among those high in intergroup disgust. Heterosexual women participants exhibited greater pleasantness and reduced disgust toward African female avatars, especially in neutral body areas—despite elevated physiological arousal in the same condition. This dissociation between explicit and implicit responses underscores the value of virtual reality in revealing subtle social dynamics. Mediation analyses were conducted in Experiment 2, where implicit racial bias (IAT) significantly predicted avatar attractiveness as a function of ethnicity — a prerequisite pathway that was absent in Experiment 1, precluding equivalent analyses in men. In women, implicit racial bias affected touch evaluations indirectly via perceived attractiveness, suggesting that implicit attitudes shape touch perception through their influence on aesthetic judgments rather than through direct appraisal of touch appropriateness or pleasantness.

Together, our findings reveal that touch is not only bodily but deeply social—modulated by interpersonal cues, internalized norms, and unconscious biases—and that immersive virtual reality offers a powerful platform to investigate and perhaps reshape these embodied social scripts.

From an evolutionary perspective, intergroup avoidance may have originated as a disease-avoidance mechanism, with disgust reactions serving to prevent both physical and symbolic contamination (Schaller & Park, 2011). Symbolized disgust can extend defensive behaviors from the biological to the social level, protecting perceived group purity and hierarchy (Rozin et al., 2009). In our studies, intergroup disgust sensitivity emerged as a key predictor of social responses. Among men, explicit intergroup disgust modulated first impressions and interpersonal distance towards opposite-gender outgroup avatars, suggesting that heightened sensitivity to potential contamination cues during intergroup interactions may translate into greater social distancing and more negative evaluation of outgroup members. Among women, implicit associations between outgroup and disgust concepts affected aesthetic judgments, which in turn predicted lower touch pleasantness during outgroup touch.

Our findings also suggest that traits related to maintenance of social hierarchy (i.e., social dominance orientation, SDO) and authoritarian attitudes (right-wing authoritarianism, RWA) can be embodied in social touch processing. Among men, conservative attitudes manifested in gendered patterns of tactile responses: higher RWA was associated with greater perceived appropriateness of opposite-sex touch, while higher SDO predicted stronger skin conductance responses to ingroup, same-gender touch compared to opposite-gender touch, possibly indicating same-gender avoidance linked to dominance concerns, in line with previous research on homophobic attitudes (Floyd, 2000). Among women, a different pattern emerged, as RWA showed no effect, whereas higher SDO predicted stronger HR deceleration during ingroup touch, suggesting increased attentional engagement. More broadly, the partial evidence that hierarchy-based and authoritarian attitudes manifest through tactile avoidance suggests physical contact may be devalued when perceived as threatening one's social position or identity status (Harper, 1985).

Overall, our findings reveal that intergroup touch implies a complex interplay between social cognition and bodily experience. Physical contact, often considered a universal form of social bonding, becomes a medium through which bias, attraction, and power asymmetries are enacted. IVR opens new avenues for studying these processes with high ecological validity while maintaining experimental control, offering a unique opportunity to examine—and ultimately mitigate—the nonverbal expression of intergroup bias. These findings are particularly relevant in the context of social immersive spaces, where virtual bodies become a central medium of social exchange (Fusaro et al., 2025). As avatars increasingly mediate identity, intimacy, and social norms, understanding how bias and cultural differences shape these embodied interactions is crucial for creating inclusive and authentic forms of social connection in digital worlds (Aglioti et al., 2025). This has implications for social IVR platforms (Sykownik et al., 2021), diversity training (Yong et al., 2024), and social-skills interventions (Salehi et al., 2025). Our findings suggest that immersive social platforms could support more comfortable intergroup interactions through adaptive social-touch design features, such as guided cooperative activities, customizable interpersonal distance boundaries, explicit touch-permission systems, and differentiated consent settings for intimate versus social body areas. At the same time, spatial comfort features — such as user-adjustable personal space boundaries — may help users anticipate and manage discomfort during intergroup contact but may also reinforce existing biases by enabling systematic avoidance of outgroup members. This calls for platforms designed to afford both personal comfort and structured intergroup contact, given converging evidence that positive virtual encounters can reduce prejudice under appropriate conditions (Tassinari et al., 2022, 2026). Finally, touches on social body areas, such as the hands, may be more appropriate for initial cross-ethnic interaction, whereas touches in other body parts perceived as intimate may require stronger contextual justification, explicit consent, and greater user control. This is especially relevant for applications aimed at prejudice reduction, diversity training, or social-skills interventions.

## 6 Limitations and future directions

Several limitations of the present study should be acknowledged. The type of relationship between the participant and the avatar administering the touch was not clarified. This ambiguity may have influenced how participants interpreted and emotionally responded to the tactile interaction. In real-life situations, touch is deeply contextual: the same gesture can be comforting, inappropriate, or neutral depending on whether it comes from a friend, a stranger, a romantic partner, or an authority figure. Without a clear relational framework, participants may have defaulted to personal assumptions or cultural norms, introducing variability in responses. In the future, it will be important to develop cover stories that can place touch within a more defined social context.

Moreover, both the participant's avatar and the touching avatars were presented in underwear in order to maximize the illusion of skin-to-skin contact among different body parts and avoid conveying additional social signals through clothing. While this approach offers experimental control, it reduces ecological validity relative to everyday clothed interactions, and future studies should examine whether the patterns observed here replicate in more typical social contexts such as workplace or public transport settings, where intergroup touch may carry different normative expectations. Although no haptic feedback was provided, previous work demonstrates that mere observation of touch applied to an embodied avatar from a first-person perspective is sufficient to elicit vicarious tactile sensations (Lisi et al., 2024; Verga et al., 2025). This visual-only approach is particularly advantageous when stimulation is delivered to multiple body areas within the same session, including intimate regions where direct haptic input would raise ethical and practical concerns. Nevertheless, future studies should consider incorporating haptic feedback, which has been shown to increase feelings of touch realism and embodiment in virtual environments (H. H. Ma et al., 2026; Richard et al., 2026; Sun et al., 2024), potentially enhancing the ecological validity of intergroup touch paradigms. Our avatars did not display dynamic facial expressions, which constitutes a potential confound, as facial cues are known to modulate the perception and affective meaning of touch (Ravaja et al., 2017). Moreover, we did not explicitly assess perceived realism or fidelity of the avatars across conditions. Given that subtle differences in rendering quality can negatively affect avatar perception (Lee & Ji, 2025), uncontrolled variation in perceived realism represents a potential confound that may have influenced participants' responses independently of the intended group membership manipulation. Future studies employing intergroup avatar paradigms — particularly on sensitive topics — should incorporate a prestudy in which candidate stimuli are rated for perceived realism.

The operationalization of ingroup and outgroup categories relied on visually defined cues (skin color and facial features) as proxies for social group membership. While this is common practice in intergroup research, it conflates racial appearance with social group identity, potentially limiting the generalizability of findings to contexts where group boundaries are defined by other criteria. Furthermore, our experiments were limited to the investigation of reactions to virtual touch from African immigrants within the Italian context. Notably, Eurobarometer data suggest that Roma and Sinti people may be subjected to even higher discrimination (https://europa.eu/eurobarometer/surveys/detail/2972; Melotti et al., 2023), and future studies should extend this paradigm to other stigmatized outgroups and cultural settings. A cautionary note regards the sample size, as the power analysis was based on a large effect size derived from a previous study using a similar paradigm that did not include an ethnicity manipulation or individual differences moderators (Fusaro et al., 2021). As such effects may be small to medium in magnitude, the current sample may have lacked sufficient power to detect them reliably. Finally, future studies could incorporate additional implicit measures of touch evaluation to minimize the influence of social desirability. These may include eye-tracking to assess visual attention patterns during touch interactions, or electroencephalography (EEG) to investigate the temporal dynamics of touch perception, including early somatosensory and affective processing.

## 7 Conclusions

Using immersive virtual reality, we have demonstrated that responses to social touch are modulated by both social evaluations of the person performing the touch (attractiveness, trustworthiness), individual differences (intergroup disgust, social dominance orientation), as well as by implicit biases that influence aesthetic judgements. The dissociation that emerged between explicit and implicit responses, particularly in the female sample, highlights the value of virtual reality as a tool for revealing subtle social dynamics. It is important to understand how biases and cultural norms shape physical contact, especially as social interactions increasingly take place in immersive virtual spaces. Our findings suggest that virtual reality social platforms should balance the need for individual spatial comfort with opportunities for structured intergroup interactions, to avoid reinforcing avoidance tendencies.

## CRediT authorship contribution statement

**Matteo P. Lisi:** Writing – review & editing, Writing – original draft, Visualization, Software, Methodology, Investigation, Formal analysis, Data curation, Conceptualization. **Anna Giometti:** Writing – review & editing, Investigation, Formal analysis, Data curation. **Martina Fusaro:** Writing – review & editing, Validation, Supervision, Methodology, Funding acquisition, Conceptualization. **Salvatore M. Aglioti:** Writing – review & editing, Supervision, Project administration, Funding acquisition, Conceptualization.

## Ethics declaration

Written informed consent to take part in the study and to publish the article has been obtained from all participants or their legal representatives. The privacy rights of participants have been observed.

This study was performed in compliance with relevant laws, regulatory frameworks and guidelines where the research took place. This study was conducted in accordance with 2013 Declaration of Helsinki. This study was approved by the LIFE Institute for Research and Health Care Santa Lucia IRCCS. (Approval No. CE/2022_012)

## Declaration of generative AI and AI-assisted technologies in the manuscript preparation process

During the preparation of this work the authors used ChatGPT as a writing assistant to support language editing and improve the readability and conceptual clarity of the text. Subsequently, the authors reviewed and edited the content as needed and take full responsibility for the content of the published article.

## Ethical statement

The experimental protocol was approved by the ethics committee of the Fondazione Santa Lucia (CE/2022_012) and was carried out in accordance with the ethical standards of the 2013 Declaration of Helsinki. All participants gave their written informed consent to take part in the study.

## Funding

This work was supported by BEFORERC, awarded by Sapienza University of Rome to M.P.L.; Brain Magnet, awarded by the Italian Institute of Technology to S.M.A.; eHONESTY, ERC Advanced Grant awarded to S.M.A. (grant number 789058).

## Declaration of Competing Interest

The authors declare that they have no known competing financial interests or personal relationships that could have appeared to influence the work reported in this paper.

## Data Availability

Raw data and analysis scripts are available at: https://figshare.com/s/477b07386822de1f7d36.
