# Supplementary material for: Surrogate bias: Behavioral and physiological effects of seeing ingroup and outgroup virtual agents touching one's embodied avatar
Source: Comput Hum Behav Rep. 2026 Aug;23:None. doi: 10.1016/j.chbr.2026.101273 (PMC13576381; doi:10.1016/j.chbr.2026.101273)
Supplement: Supplementary file 1 [file mmc1.docx]

**Supplementary Information for**

**Surrogate bias: Behavioral and physiological effects of seeing ingroup and outgroup virtual agents touching one’s embodied avatar**

Matteo P. Lisi^1,2*^, Anna Giometti^1,2,3^, Martina Fusaro^2,4^, Salvatore M. Aglioti^1,2*^

1 CLN2S@Sapienza, Fondazione Istituto Italiano di Tecnologia (IIT) and Dipartimento di Psicologia, Università La Sapienza, Rome, Italy

2 IRCCS Fondazione Santa Lucia, Rome, Italy.

3 School of Advanced Studies, Centre for Neuroscience, University of Camerino, Camerino, Italy

4 Consiglio Nazionale delle Ricerche (CNR), Istituto di Scienze e Tecnologie della Cognizione, Rome, Italy.

**Supplementary Materials**

Supplementary video for the interpersonal distance task, along with the data and the analysis code are available at the following link: <https://figshare.com/s/477b07386822de1f7d36>

**Fig. S1** The four avatars used for the virtual touch and the interpersonal distance task.

**Tab. S1**. In-session and end-of-block VAS questions.

| Category | Question/statement |
| --- | --- |
| Appropriateness | How appropriate was the touch? |
| Pleasantness | How pleasant was the touch? |
| Disgust | How disgusting was the touch? |
| Erogeneity | How erogenous was the touch? |
| Ownership | “It seemed like I was watching my body” |
| Ownership | “It seemed like the virtual body was my body” |
| Ownership (control) | “It seemed like I had more than one body” |
| Ownership (control) | “It seemed like I did not have my body anymore” |
| Vicarious touch | “It seemed like I was feeling the touches on my body” |
| Attractiveness | How attractive was the avatar? |
| Trustworthiness | How trustworthy was the avatar? |

**Tonic Physiological Arousal**

To compute heart rate variability (HRV), interbeat intervals were written in a text file, and later analyzed using Kubios HRV software (Tarvainen et al.,2014). The baseline-corrected root mean square of successive beat-to-beat interval differences (RMSSD), High-Frequency (HF) band values, and Sample Entropy (SampEn) entered the analyses as dependent variables, with the interaction between Avatar Gender and avatarethnicity as predictors. Baseline correction was based on the 1-minute resting period before the start of the virtual touch task. Skin conductance level was computed using Ledalab (Benedek & Kaembach, 2010).

**Supplementary Results**

**Experiment 1: Caucasian heterosexual men**

**RATINGS OF AVATARS’ FEATURES**

**Attractiveness**
Participants evaluated the attractiveness of each avatar (see Supplemental Information for the exact wording of the question). The analysis (R^2^marginal = 0.52, R^2^conditional = 0.56) revealed a main effect of Avatar Gender (χ²(1) = 205.69, *p* < .001), with female avatars rated as significantly more attractive (mean = 72, *SE* = 2.65) than male avatars (mean = 22, *SE* = 2.65; *b* = 49, *SE* = 3.5, *t* = 14.30, *p* < .0001). No significant main effect of Avatar Ethnicity (χ²(1) = 1.89, p = .17) or Avatar Gender × Avatar Ethnicity interaction (χ²(1) = 0.10, *p* = .75) was found. Importantly, a significant three-way interaction between Avatar Gender, Avatar Ethnicity, and intergroup disgust sensitivity emerged (χ²(4) = 11.66, *p* = .02). Trend analyses indicated that higher intergroup disgust sensitivity scores were associated with reduced attractiveness ratings specifically for African female avatars (*b* = –10.4, *SE* = 3.7). No other significant results were found.

**Tab.S3** Model comparison for predictors of Attractiveness in men. Candidate mixed-effects models were compared using maximum likelihood estimation and ranked by AIC. The best fitting model (lowest AIC) included avatargender, avatarethnicity, and their interaction with intergroup disgust sensitivity (itg). χ² tests and corresponding p-values indicate improvements over nested models.

| **Formula** | **npar** | **AIC** | **BIC** | **logLik** |
| --- | --- | --- | --- | --- |
| **Attractiveness ~ avatargender * avatarethnicity + avatargender:avatarethnicity:itg_sc + (1 \| ID)** | **10** | **1790.5** | **1823.07** | **-885.25** |
| Attractiveness ~ avatargender * avatarethnicity + avatargender:rwa_sc +  (1 \| ID) | 8 | 1792.22 | 1818.28 | -888.11 |
| Attractiveness ~ avatargender * avatarethnicity + (1 \| ID) | 6 | 1793.9 | 1813.44 | -890.95 |
| Attractiveness ~ avatargender * avatarethnicity + avatarethnicity:iat_sc + (1 \| ID) | 8 | 1794.55 | 1820.61 | -889.28 |
| Attractiveness ~ avatargender * avatarethnicity + avatargender:sdo_sc +  (1 \| ID) | 8 | 1795.09 | 1821.15 | -889.55 |
| Attractiveness ~ avatargender * avatarethnicity + avatargender:avatarethnicity:rwa_sc + (1 \| ID) | 10 | 1796.06 | 1828.63 | -888.03 |
| Attractiveness ~ avatargender * avatarethnicity + avatargender:avatarethnicity:iat_sc + (1 \| ID) | 10 | 1796.07 | 1828.64 | -888.03 |
| Attractiveness ~ avatargender * avatarethnicity + avatargender:avatarethnicity:sdo_sc + (1 \| ID) | 10 | 1799.07 | 1831.65 | -889.54 |

**Trustworthiness**

The linear mixed-effects model (R^2^marginal = 0.17, R^2^conditional = 0.58) revealed a significant main effect of Avatar Gender (χ²(1) = 34.37, *p* < .001), indicating that participants rated female avatars (mean = 61, *SE* = 3.06) as more trustworthy than male avatars (mean = 47, *SE* = 3.06; *b* = 14.6, *SE* = 2.47, *t* = 5.90, *p* < .0001). No effect of Avatar Ethnicity was also found (χ²(1) = 3.86, *p* = .05), though no significant interaction between gender and ethnicity emerged (χ²(1) = 0.05, *p* = .82). Importantly, a significant three-way interaction between Avatar Gender, Ethnicity, and intergroup disgust sensitivity scores was observed (χ²(4) = 13.25, *p* = .01). Trend analyses revealed that trustworthiness ratings for male African avatars decreased as intergroup disgust sensitivity increased (*b* = –12.3, *SE* = 3.6). These findings suggest that higher intergroup disgust sensitivity is associated with reduced perceived trustworthiness of same-gender outgroup avatars.

**Tab.S4** Model comparison for predictors of Trustworthiness in men. Candidate mixed-effects models were compared using maximum likelihood estimation and ranked by AIC. The best fitting model (lowest AIC) included avatargender, avatarethnicity, and the interaction between Touching Avatar Gender and Right Wing Authoritarianism. χ² tests and corresponding p-values indicate improvements over nested models.

| **Formula** | **npar** | **AIC** | **BIC** | **logLik** |
| --- | --- | --- | --- | --- |
| **Trustworthiness ~ avatargender * avatarethnicity + avatargender:rwa_sc + (1 \| ID)** | **8** | **1724.17** | **1750.23** | **-854.08** |
| Trustworthiness ~ avatargender * avatarethnicity + avatargender:avatarethnicity:itg_sc + (1 \| ID) | 10 | 1725.79 | 1758.37 | -852.9 |
| Trustworthiness ~ avatargender * avatarethnicity + avatargender:sdo_sc + (1 \| ID) | 8 | 1725.8 | 1751.86 | -854.9 |
| Trustworthiness ~ avatargender * avatarethnicity + avatargender:avatarethnicity:rwa_sc + (1 \| ID) | 10 | 1727.96 | 1760.53 | -853.98 |
| Trustworthiness ~ avatargender * avatarethnicity + avatargender:avatarethnicity:sdo_sc + (1 \| ID) | 10 | 1728.57 | 1761.14 | -854.28 |
| Trustworthiness ~ avatargender * avatarethnicity + (1 \| ID) | 6 | 1731.05 | 1750.6 | -859.53 |
| Trustworthiness ~ avatargender * avatarethnicity + avatarethnicity:iat_sc + (1 \| ID) | 8 | 1734.74 | 1760.8 | -859.37 |
| Trustworthiness ~ avatargender * avatarethnicity + avatargender:avatarethnicity:iat_sc + (1 \| ID) | 10 | 1738.23 | 1770.81 | -859.12 |

**VIRTUAL TOUCH TASK**

**Appropriateness of touch**

**Tab.S5** Model comparison for predictors of Appropriateness in heterosexual caucasian men. Candidate mixed-effects models were compared using maximum likelihood estimation and ranked by AIC. The best fitting model (lowest AIC) included the effects of Attractiveness, Trustworthiness, and the interaction between Touching Avatar Gender and Right Wing Authoritarianism.

| **Formula** | **npar** | | **AIC** | **BIC** | **logLik** |
| --- | --- | --- | --- | --- | --- |
| Appropriateness ~ avatargender * avatarethnicity * area + Attractiveness_sc + Trustworthiness_sc + avatargender:rwa_sc + (1 + avatargender + avatarethnicity + area \| ID) | | 32 | 20452.7 | 20636.46 | -10194.35 |
| Appropriateness ~ avatargender * avatarethnicity * area + Attractiveness_sc + Trustworthiness_sc + avatarethnicity:area:iat_sc + (1 + avatargender + ethnicity + area \| ID) | | 36 | 20454.07 | 20660.8 | -10191.03 |
| Appropriateness ~ avatargender * avatarethnicity * area + Attractiveness_sc + Trustworthiness_sc + avatargender:avatarethnicity:rwa_sc + (1 + avatargender + avatarethnicity + area \| ID) | | 34 | 20455.07 | 20650.31 | -10193.53 |
| Appropriateness ~ avatargender * avatarethnicity * area + Attractiveness_sc + Trustworthiness_sc + avatargender:avatarethnicity:iat_sc + (1 + avatargender + avatarethnicity + area \| ID) | | 34 | 20461.05 | 20656.29 | -10196.53 |
| Appropriateness ~ avatargender * avatarethnicity * area + Attractiveness_sc + Trustworthiness_sc + avatarethnicity:area:rwa_sc + (1 + avatargender + ethnicity + area \| ID) | | 36 | 20463.9 | 20670.63 | -10195.95 |
| Appropriateness ~ avatargender * avatarethnicity * area + Attractiveness_sc + Trustworthiness_sc + avatargender:sdo_sc + (1 + avatargender + avatarethnicity + area \| ID) | | 32 | 20463.9 | 20647.66 | -10199.95 |
| Appropriateness ~ avatargender * avatarethnicity * area + Attractiveness_sc + Trustworthiness_sc + (1 + avatargender + avatarethnicity + area \| ID) | | 30 | 20464.37 | 20636.64 | -10202.19 |
| Appropriateness ~ avatargender * avatarethnicity * area + Trustworthiness_sc + (1 + avatargender + avatarethnicity + area \| ID) | | 29 | 20464.7 | 20631.23 | -10203.35 |
| Appropriateness ~ avatargender * avatarethnicity * area + Attractiveness_sc + Trustworthiness_sc + avatargender:avatarethnicity:sdo_sc + (1 + avatargender + avatarethnicity + area \| ID) | | 34 | 20466.06 | 20661.3 | -10199.03 |
| Appropriateness ~ avatargender * avatarethnicity * area + Attractiveness_sc + Trustworthiness_sc + avatarethnicity:area:sdo_sc + (1 + avatargender + ethnicity + area \| ID) | | 36 | 20466.71 | 20673.44 | -10197.36 |
| Appropriateness ~ avatargender * avatarethnicity * area + Attractiveness_sc + Trustworthiness_sc + avatargender:avatarethnicity:itg_sc + (1 + avatargender + avatarethnicity + area \| ID) | | 34 | 20468.16 | 20663.4 | -10200.08 |
| Appropriateness ~ avatargender * avatarethnicity * area + Attractiveness_sc + (1 + avatargender + avatarethnicity + area \| ID) | | 29 | 20469.32 | 20635.85 | -10205.66 |
| Appropriateness ~ avatargender * avatarethnicity * area + Attractiveness_sc + Trustworthiness_sc + avatarethnicity:area:itg_sc + (1 + avatargender + ethnicity + area \| ID) | | 36 | 20473.4 | 20680.12 | -10200.7 |
| Appropriateness ~ avatargender * avatarethnicity * area + (1 + avatargender + ethnicity + area \| ID) | | 28 | 20477.45 | 20638.24 | -10210.72 |
| Appropriateness ~ avatargender * avatarethnicity * area + stq_sc + (1 + avatargender + avatarethnicity + area \| ID) | | 29 | 20478.09 | 20644.62 | -10210.05 |

**Pleasantness of touch**

**Tab.S6** Model comparison for predictors of Pleasantness in heterosexual caucasian men. Candidate mixed-effects models were compared using maximum likelihood estimation and ranked by AIC. The best fitting model (lowest AIC) included the effects of Attractiveness, Trustworthiness, and the interaction between Touching Avatar Gender and Right Wing Authoritarianism.

| **Formula** | **npar** | **AIC** | **BIC** | **logLik** |
| --- | --- | --- | --- | --- |
| Pleasantness ~ avatargender * avatarethnicity * area + Attractiveness_sc + Trustworthiness_sc + (1 + avatargender + avatarethnicity \| ID) | 21 | 19716.37 | 19836.96 | -9837.18 |
| Pleasantness ~ avatargender * avatarethnicity * area + Attractiveness_sc + Trustworthiness_sc + avatargender:rwa_sc + (1 + avatargender + avatarethnicity \| ID) | 23 | 19716.95 | 19849.03 | -9835.47 |
| Pleasantness ~ avatargender * avatarethnicity * area + Attractiveness_sc + Trustworthiness_sc + avatargender:avatarethnicity:rwa_sc + (1 + avatargender + avatarethnicity \| ID) | 25 | 19717.01 | 19860.57 | -9833.51 |
| Pleasantness ~ avatargender * avatarethnicity * area + Attractiveness_sc + Trustworthiness_sc + avatargender:avatarethnicity:itg_sc + (1 + avatargender + avatarethnicity \| ID) | 25 | 19717.05 | 19860.61 | -9833.53 |
| Pleasantness ~ avatargender * avatarethnicity * area + Attractiveness_sc + Trustworthiness_sc + avatargender:avatarethnicity:sdo_sc + (1 + avatargender + avatarethnicity \| ID) | 25 | 19717.92 | 19861.48 | -9833.96 |
| Pleasantness ~ avatargender * avatarethnicity * area + Attractiveness_sc + (1 + avatargender + avatarethnicity \| ID) | 20 | 19719.28 | 19834.13 | -9839.64 |
| Pleasantness ~ avatargender * avatarethnicity * area + Attractiveness_sc + Trustworthiness_sc + avatargender:avatarethnicity:iat_sc + (1 + avatargender + avatarethnicity \| ID) | 25 | 19719.86 | 19863.42 | -9834.93 |
| Pleasantness ~ avatargender * avatarethnicity * area + Attractiveness_sc + Trustworthiness_sc + avatargender:sdo_sc + (1 + avatargender + avatarethnicity \| ID) | 23 | 19719.99 | 19852.07 | -9837 |
| Pleasantness ~ avatargender * avatarethnicity * area + Trustworthiness_sc + (1 + avatargender + avatarethnicity \| ID) | 20 | 19729.27 | 19844.12 | -9844.63 |
| Pleasantness ~ avatargender * avatarethnicity * area + (1 + avatargender + avatarethnicity \| ID) | 19 | 19746.18 | 19855.29 | -9854.09 |
| Pleasantness ~ avatargender * avatarethnicity * area + stq_sc + (1 + avatargender + avatarethnicity \| ID) | 20 | 19748.14 | 19862.99 | -9854.07 |

**Disgust of touch**

The linear mixed-effects model (R^2^marginal = 0.076, R^2^conditional = 0.53) revealed a significant main effect of Attractiveness (χ²(1) = 10.98, p < .001), with higher attractiveness associated with lower levels of disgust toward the avatars (β = 3.08, SE=1.20, *t*= 2.56). A significant interaction also emerged between Avatar Gender and Area (χ²(2) = 35.38, *p* < .001): in the intimate area, participants reported significantly less disgust when touched by female avatars compared to male avatars (*b* = -9.60, SE = 4.54, t= -2.11, *p* = .038, *d*=-0.49). No gender differences were found in the neutral or social areas.

**Tab.S7** Model comparison for predictors of Disgust in heterosexual caucasian men. Candidate mixed-effects models were compared using maximum likelihood estimation and ranked by AIC. The best fitting model (lowest AIC) included the effects of Attractiveness, and the interaction between Touching Avatar Gender and Social Dominance Orientation.

| **Formula** | **npar** | **AIC** | **BIC** | **logLik** |
| --- | --- | --- | --- | --- |
| Disgust ~ avatargender * avatarethnicity * area + Attractiveness_sc + avatargender:sdo_sc + (1 + avatargender + avatarethnicity \| ID) | 22 | 20683.34 | 20809.68 | -10319.67 |
| Disgust ~ avatargender * avatarethnicity * area + Attractiveness_sc + avatargender:avatarethnicity:sdo_sc + (1 + avatargender + avatarethnicity \| ID) | 24 | 20684.28 | 20822.1 | -10318.14 |
| Disgust ~ avatargender * avatarethnicity * area + Attractiveness_sc + (1 + avatargender + avatarethnicity \| ID) | 20 | 20685.02 | 20799.86 | -10322.51 |
| Disgust ~ avatargender * avatarethnicity * area + Attractiveness_sc + avatargender:avatarethnicity:itg_sc + (1 + avatargender + avatarethnicity \| ID) | 24 | 20685.24 | 20823.06 | -10318.62 |
| Disgust ~ avatargender * avatarethnicity * area + Attractiveness_sc + avatargender:rwa_sc + (1 + avatargender + avatarethnicity \| ID) | 22 | 20685.73 | 20812.06 | -10320.86 |
| Disgust ~ avatargender * avatarethnicity * area + Attractiveness_sc + avatargender:avatarethnicity:iat_sc + (1 + avatargender + avatarethnicity \| ID) | 24 | 20686.53 | 20824.34 | -10319.26 |
| Disgust ~ avatargender * avatarethnicity * area + Attractiveness_sc + Trustworthiness_sc + (1 + avatargender + avatarethnicity \| ID) | 21 | 20686.72 | 20807.31 | -10322.36 |
| Disgust ~ avatargender * avatarethnicity * area + Attractiveness_sc + avatargender:avatarethnicity:rwa_sc + (1 + avatargender + avatarethnicity \| ID) | 24 | 20689.29 | 20827.1 | -10320.64 |
| Disgust ~ avatargender * avatarethnicity * area + stq_sc + (1 + avatargender + avatarethnicity \| ID) | 20 | 20693.27 | 20808.11 | -10326.63 |
| Disgust ~ avatargender * avatarethnicity * area + (1 + avatargender + avatarethnicity \| ID) | 19 | 20693.29 | 20802.4 | -10327.65 |
| Disgust ~ avatargender * avatarethnicity * area + Trustworthiness_sc + (1 + avatargender + avatarethnicity \| ID) | 20 | 20694.25 | 20809.1 | -10327.12 |

**Erogeneity of touch**

**Tab.S8** Model comparison for predictors of Erogeneity in heterosexual caucasian men. Candidate mixed-effects models were compared using maximum likelihood estimation and ranked by AIC. The best fitting model (lowest AIC) included the effects of Attractiveness, and the interaction between Touching Avatar Gender and Social Dominance Orientation.

| **Formula** | **npar** | **AIC** | **BIC** | **logLik** |
| --- | --- | --- | --- | --- |
| Erogeneity ~ avatargender * avatarethnicity * area + Attractiveness_sc + Trustworthiness_sc + avatargender:sdo_sc + (1 + avatargender + avatarethnicity \| ID) | 23 | 20562.36 | 20694.44 | -10258.18 |
| Erogeneity ~ avatargender * avatarethnicity * area + Attractiveness_sc + Trustworthiness_sc + (1 + avatargender + avatarethnicity \| ID) | 21 | 20563.9 | 20684.49 | -10260.95 |
| Erogeneity ~ avatargender * avatarethnicity * area + Attractiveness_sc + Trustworthiness_sc + avatargender:avatarethnicity:sdo_sc + (1 + avatargender + avatarethnicity \| ID) | 25 | 20564.44 | 20708 | -10257.22 |
| Erogeneity ~ avatargender * avatarethnicity * area + Attractiveness_sc + Trustworthiness_sc + avatargender:rwa_sc + (1 + avatargender + avatarethnicity \| ID) | 23 | 20564.97 | 20697.04 | -10259.48 |
| Erogeneity ~ avatargender * avatarethnicity * area + Attractiveness_sc + Trustworthiness_sc + avatargender:avatarethnicity:rwa_sc + (1 + avatargender + avatarethnicity \| ID) | 25 | 20567.09 | 20710.65 | -10258.54 |
| Erogeneity ~ avatargender * avatarethnicity * area + Attractiveness_sc + Trustworthiness_sc + avatargender:avatarethnicity:iat_sc + (1 + avatargender + avatarethnicity \| ID) | 25 | 20567.97 | 20711.53 | -10258.99 |
| Erogeneity ~ avatargender * avatarethnicity * area + Attractiveness_sc + Trustworthiness_sc + avatargender:avatarethnicity:itg_sc + (1 + avatargender + avatarethnicity \| ID) | 25 | 20569.04 | 20712.6 | -10259.52 |
| Erogeneity ~ avatargender * avatarethnicity * area + Trustworthiness_sc + (1 + avatargender + avatarethnicity \| ID) | 20 | 20570.79 | 20685.64 | -10265.4 |
| Erogeneity ~ avatargender * avatarethnicity * area + Attractiveness_sc + (1 + avatargender + avatarethnicity \| ID) | 20 | 20576.35 | 20691.2 | -10268.18 |
| Erogeneity ~ avatargender * avatarethnicity * area + stq_sc + (1 + avatargender + avatarethnicity \| ID) | 20 | 20604.68 | 20719.53 | -10282.34 |
| Erogeneity ~ avatargender * avatarethnicity * area + (1 + avatargender + avatarethnicity \| ID) | 19 | 20605.11 | 20714.21 | -10283.55 |

**PHYSIOLOGICAL REACTIVITY TO TOUCH**

**Skin Conductance Responses**

**Tab.S9** Model comparison for predictors of SCR in heterosexual caucasian men. Candidate mixed-effects models were compared using maximum likelihood estimation and ranked by AIC. The best fitting model (lowest AIC) included the effects of Attractiveness, and the interaction between Touching Avatar Gender and Social Dominance Orientation.

| **Formula** | **npar** | **AIC** | **BIC** | **logLik** |
| --- | --- | --- | --- | --- |
| log_scr ~ avatargender * avatarethnicity * area + stq_sc + avatargender:avatarethnicity :sdo_sc + (1 + avatargender + avatarethnicity \| ID) | 24 | 6090.18 | 6227.88 | -3021.09 |
| log_scr ~ avatargender * avatarethnicity * area + stq_sc + avatargender:rwa_sc + (1 + avatargender + avatarethnicity \| ID) | 22 | 6091.7 | 6217.92 | -3023.85 |
| log_scr ~ avatargender * avatarethnicity * area + stq_sc + (1 + avatargender + avatarethnicity \| ID) | 20 | 6094.82 | 6209.57 | -3027.41 |
| log_scr ~ avatargender * avatarethnicity * area + stq_sc + avatargender:avatarethnicity:iat_sc + (1 + avatargender + avatarethnicity \| ID) | 24 | 6095.6 | 6233.3 | -3023.8 |
| log_scr ~ avatargender * avatarethnicity * area + stq_sc + avatargender:sdo_sc + (1 + avatargender + avatarethnicity \| ID) | 22 | 6095.87 | 6222.1 | -3025.94 |
| log_scr ~ avatargender * avatarethnicity * area + stq_sc + avatargender:avatarethnicity:rwa_sc + (1 + avatargender + avatarethnicity + area \| ID) | 33 | 6096.26 | 6285.6 | -3015.13 |
| log_scr ~ avatargender * avatarethnicity * area + stq_sc + avatargender:avatarethnicity:itg_sc + (1 + avatargender + avatarethnicity \| ID) | 24 | 6099.25 | 6236.95 | -3025.62 |

**Heart Rate**

**Tab.S10** Model comparison for predictors of HR in heterosexual caucasian men.

| **Formula** | **npar** | **AIC** | **BIC** | **logLik** |
| --- | --- | --- | --- | --- |
| hr ~ avatargender * avatarethnicity * area + Attractiveness_sc + (1 + avatargender + avatarethnicity \| ID) | 20 | 12168.55 | 12282.36 | -6064.27 |
| hr ~ avatargender * avatarethnicity * area + Attractiveness_sc + avatargender:avatarethnicity:itg_sc + (1 + avatargender + avatarethnicity \| ID) | 24 | 12169.18 | 12305.75 | -6060.59 |
| hr ~ avatargender * avatarethnicity * area + Attractiveness_sc + avatargender:sdo_sc + (1 + avatargender + avatarethnicity \| ID) | 22 | 12170.74 | 12295.92 | -6063.37 |
| hr ~ avatargender * avatarethnicity * area + Attractiveness_sc + avatargender:rwa_sc + (1 + avatargender + avatarethnicity \| ID) | 22 | 12171.54 | 12296.73 | -6063.77 |
| hr ~ avatargender * avatarethnicity * area + Attractiveness_sc + avatargender:avatarethnicity:sdo_sc + (1 + avatargender + avatarethnicity \| ID) | 24 | 12173.2 | 12309.77 | -6062.6 |
| hr ~ avatargender * avatarethnicity * area + Attractiveness_sc + avatargender:avatarethnicity:iat_sc + (1 + avatargender + avatarethnicity \| ID) | 24 | 12173.5 | 12310.06 | -6062.75 |
| hr ~ avatargender * avatarethnicity * area + Attractiveness_sc + avatargender:avatarethnicity:rwa_sc + (1 + avatargender + avatarethnicity + area \| ID) | 33 | 12184.69 | 12372.47 | -6059.35 |

**Skin conductance Level (SCL)**

The model (R² marginal = 0.0039, R² conditional = 0.0039) did not show any significant modulation of this measure in relation to the avatar’s gender, avatarethnicity, or participants’ scores on the Social Touch Questionnaire (STQ) (all ps > .65; see Table Sx). The interaction between avatar gender and avatarethnicity was also not significant (*p* = .50), suggesting that tonic skin conductance levels were not influenced by the experimental manipulations nor by individual differences in social touch attitudes.

**Heart Rate Variability (HRV)**

None of the measures (RMSSD, HF, SampleEn) showed significant results.

**Interpersonal Distance**

**Tab.S11** Model comparison for predictors of IPD in heterosexual caucasian men.

| **Formula** | **npar** | **AIC** | **BIC** | **logLik** |
| --- | --- | --- | --- | --- |
| distance ~ avatargender * avatarethnicity + Trustworthiness_sc + avatargender:ethnicity:itg_sc + (1 \| ID) | 11 | 1831.07 | 1866.90 | -904.53 |
| distance ~ avatargender * avatarethnicity + Trustworthiness_sc + avatargender:rwa_sc + (1 \| ID) | 9 | 1836.23 | 1865.55 | -909.11 |
| distance ~ avatargender * avatarethnicity + Trustworthiness_sc + avatargender:sdo_sc + (1 \| ID) | 9 | 1838.25 | 1867.57 | -910.12 |
| distance ~ avatargender * avatarethnicity + Trustworthiness_sc + avatargender:avatarethnicity:rwa_sc + (1 \| ID) | 11 | 1839.12 | 1874.95 | -908.56 |
| distance ~ avatargender * avatarethnicity + Trustworthiness_sc + avatargender:avatarethnicity:sdo_sc + (1 \| ID) | 7 | 1840.64 | 1876.48 | -909.32 |
| distance ~ avatargender * avatarethnicity + Trustworthiness_sc + (1 \| ID) | 11 | 1843.52 | 1866.32 | -914.76 |
| distance ~ avatargender * avatarethnicity + Trustworthiness_sc + avatargender:avatarethnicity:iat_sc + (1 \| ID) | 11 | 1847.65 | 1883.48 | -912.82 |

**Body ownership questionnaire**

The two embodiment questions and the two control questions were averaged to create an embodiment score for each condition. The linear mixed model (R^2^marginal = 0.37, R^2^conditional = 0.85) revealed a significant main effect of the Question factor (χ²(1) = 75.11, *p* < 0.001), with higher ratings for the embodiment items (mean 62, SE 2.87,) compared to the control items (mean = 29, *SE* = 2.86; *b* = -33, *SE* = 3.9, *t* = -8.70, *p* < 0.001), suggesting that participants experienced a sense of body ownership over the virtual avatar. No other interactions reached significance.

**Vicarious touch**

The analysis of subjective reports concerning the illusory sensation of being touched on one’s own body (item 3, see Supplementary Information, R²marginal = 0.014, R²conditional = 0.7) revealed a significant effect of Avatar Gender (χ²(1) = 4.26, *p* = 0.039), with a slightly higher level of illusion during female touch (*b* = 4.2, *SE* = 2.04, *t* = 2.06, p = 0.041) compared to male touch (mean for female-touching avatar = 49, *SE* = 3.4 vs. mean for male-touching avatar = 45, *SE* = 3.4). The interaction between Avatar Gender and Avatar avatarethnicity (χ²(1) = 4.45, *p* = 0.035), showed that the level of illusion was higher during the outgroup female touch (mean 51, SE = 3.7) compared to the outgroup male touch (mean = 43, *SE* = 3.7); *b* = 8.5, *SE* = 2.88, *t* = 2.95).

**Experiment 2: Caucasian heterosexual women**

**RATINGS OF AVATARS’ FEATURES**

**Attractiveness**

The linear mixed-effects model (R^2^marginal = .09, R^2^conditional = .35) revealed a significant Avatar Gender by Avatar Ethnicity interaction (χ²(1) = 7.61, *p* = .006), indicating that the perceived attractiveness of the avatars varied depending on the combination of their gender and ethnicity. Specifically, male African avatars were rated as significantly more attractive than male Caucasian avatars (*b* =16.12, *SE* = 4.95, *t* = 3.26, *p* = .001).
Furthermore, a significant three-way interaction between Avatar Gender, Avatar Ethnicity, and Implicit Association Test was observed (χ²(4) = 11.98, p = .017), suggesting that implicit racial bias (as measured by the IAT) influenced attractiveness ratings. This effect was particularly evident for male avatars, with a significant difference between African and Caucasian avatars (*b* = -13.75, *SE* 5.34, *t* = -2.57, *p* = .011), indicating that higher IAT scores (i.e., higher implicit bias) were associated with lower attractiveness ratings for African male avatars. This pattern supports the notion that implicit biases can impact perceptions of attractiveness, particularly for outgroup members.

**Tab.S11** Model comparison for predictors of Attractiveness in heterosexual caucasian women. Candidate mixed-effects models were compared using maximum likelihood estimation and ranked by AIC. The best fitting model (lowest AIC) included avatargender, avatarethnicity, and their interaction wit h the d score obtained from the implicit association test (IAT).

| **Formula** | **npar** | **AIC** | **BIC** | **logLik** |
| --- | --- | --- | --- | --- |
| Attractiveness ~ avatargender * avatarethnicity + avatargender:avatarethnicity:iat_sc + (1 \| ID) | 10 | 1826.61 | 1859.19 | -903.31 |
| Attractiveness ~ avatargender * avatarethnicity + avatarethnicity:iat_sc + (1 \| ID) | 8 | 1827.43 | 1853.49 | -905.72 |
| Attractiveness ~ avatargender * avatarethnicity + avatargender:rwa_sc + (1 \| ID) | 8 | 1828.45 | 1854.51 | -906.23 |
| Attractiveness ~ 1 + avatargender * avatarethnicity + (1 \| ID) | 6 | 1830.72 | 1850.26 | -909.36 |
| Attractiveness ~ avatargender * avatarethnicity + avatargender:avatarethnicity:rwa_sc + (1 \| ID) | 10 | 1831.84 | 1864.42 | -905.92 |
| Attractiveness ~ avatargender * avatarethnicity + avatargender:avatarethnicity:itg_sc + (1 \| ID) | 10 | 1832.98 | 1865.55 | -906.49 |
| Attractiveness ~ avatargender * avatarethnicity + avatargender:sdo_sc + (1 \| ID) | 8 | 1834.55 | 1860.61 | -909.27 |
| Attractiveness ~ avatargender * avatarethnicity + avatargender:avatarethnicity:sdo_sc + (1 \| ID) | 10 | 1835.38 | 1867.96 | -907.69 |

**Trustworthiness**

The linear mixed-effects model (R^2^marginal = .084, R^2^conditional = .68) revealed significant main effects of Avatar Gender (χ²(1) = 19.40, *p* < .0001) and Avatar Ethnicity (χ²(1) = 16.51, *p* < .0001) on trustworthiness ratings. Female avatars were perceived as significantly more trustworthy than male avatars (*b* = 9.25, *SE* = 2.1, *p* < .0001), and African avatars were rated as more trustworthy than Caucasian avatars (*b* = 8.53, *SE* = 2.1, *p* = .0001).
Additionally, a significant interaction between Avatar Ethnicity and IAT scores was observed (χ²(2) = 9.37, *p* = .009), indicating that higher levels of implicit bias are associated with a greater reduction in trustworthiness ratings for African avatars compared to Caucasian avatars (*b* = -6.76, *SE* = 2.27, *p* = 0.003).

**Tab.S12** Model comparison for predictors of Trustworthiness in heterosexual caucasian men. Candidate mixed-effects models were compared using maximum likelihood estimation and ranked by AIC. The best fitting model (lowest AIC) included avatargender, avatarethnicity, and the interaction between Touching Avatar Gender and Right Wing Authoritarianism.

| **Formula** | **npar** | **AIC** | **BIC** | **logLik** |
| --- | --- | --- | --- | --- |
| Trustworthiness ~ avatargender * avatarethnicity + avatarethnicity:iat_sc + (1 \| ID) | 8 | 1686.14 | 1712.2 | -835.07 |
| Trustworthiness ~ avatargender * avatarethnicity + avatargender:avatarethnicity:iat_sc + (1 \| ID) | 10 | 1688.89 | 1721.47 | -834.45 |
| Trustworthiness ~ avatargender * avatarethnicity + avatargender:avatarethnicity:rwa_sc + (1 \| ID) | 10 | 1691.28 | 1723.86 | -835.64 |
| Trustworthiness ~ avatargender * avatarethnicity + avatargender:rwa_sc + (1 \| ID) | 8 | 1691.5 | 1717.56 | -837.75 |
| Trustworthiness ~ 1 + avatargender * avatarethnicity + (1 \| ID) | 6 | 1691.51 | 1711.05 | -839.75 |
| Trustworthiness ~ avatargender * avatarethnicity + avatargender:avatarethnicity:itg_sc + (1 \| ID) | 10 | 1693.67 | 1726.24 | -836.83 |
| Trustworthiness ~ avatargender * avatarethnicity + avatargender:sdo_sc + (1 \| ID) | 8 | 1695.38 | 1721.44 | -839.69 |
| Trustworthiness ~ avatargender * avatarethnicity + avatargender:avatarethnicity:sdo_sc + (1 \| ID) | 10 | 1697.23 | 1729.81 | -838.62 |

**VIRTUAL TOUCH TASK**

**Appropriateness of touch**

**Tab.S13** Model comparison for predictors of Appropriateness in heterosexual caucasian women. Candidate mixed-effects models were compared using maximum likelihood estimation and ranked by AIC. The best fitting model (lowest AIC) included the effects of Attractiveness and Trustworthiness.

| **Formula** | **npar** | **AIC** | **BIC** | **logLik** |
| --- | --- | --- | --- | --- |
| Appropriateness ~ avatargender * avatarethnicity * area + Attractiveness_sc + Trustworthiness_sc + (1 + avatargender + avatarethnicity + area \| ID) | 30 | 19905.81 | 20078 | -9922.91 |
| Appropriateness ~ avatargender * avatarethnicity * area + Attractiveness_sc + (1 + avatargender + avatarethnicity + area \| ID) | 29 | 19906.23 | 20072.67 | -9924.12 |
| Appropriateness ~ avatargender * avatarethnicity * area + Attractiveness_sc + avatargender:sdo_sc + (1 + avatargender + avatarethnicity + area \| ID) | 31 | 19908.71 | 20086.63 | -9923.36 |
| Appropriateness ~ avatargender * avatarethnicity * area + Attractiveness_sc + avatargender:rwa_sc + (1 + avatargender + avatarethnicity + area \| ID) | 31 | 19908.86 | 20086.78 | -9923.43 |
| Appropriateness ~ avatargender * avatarethnicity * area + Attractiveness_sc + avatargender:avatarethnicity:itg_sc + (1 + avatargender + avatarethnicity + area \| ID) | 33 | 19910.1 | 20099.5 | -9922.05 |
| Appropriateness ~ avatargender * avatarethnicity * area + Attractiveness_sc + avatarethnicity:area:sdo_sc + (1 + avatargender + avatarethnicity + area \| ID) | 35 | 19910.12 | 20111 | -9920.06 |
| Appropriateness ~ avatargender * avatarethnicity * area + Attractiveness_sc + avatargender:avatarethnicity:iat_sc + (1 + avatargender + avatarethnicity + area \| ID) | 33 | 19910.94 | 20100.34 | -9922.47 |
| Appropriateness ~ avatargender * avatarethnicity * area + Trustworthiness_sc + (1 + avatargender + avatarethnicity + area \| ID) | 29 | 19911.22 | 20077.66 | -9926.61 |
| Appropriateness ~ avatargender * avatarethnicity * area + Attractiveness_sc + avatarethnicity:area:iat_sc + (1 + avatargender + avatarethnicity + area \| ID) | 35 | 19911.34 | 20112.22 | -9920.67 |
| Appropriateness ~ avatargender * avatarethnicity * area + Attractiveness_sc + avatarethnicity:area:itg_sc + (1 + avatargender + avatarethnicity + area \| ID) | 35 | 19911.62 | 20112.5 | -9920.81 |
| Appropriateness ~ avatargender * avatarethnicity * area + Attractiveness_sc + avatarethnicity:area:rwa_sc + (1 + avatargender + avatarethnicity + area \| ID) | 35 | 19911.65 | 20112.53 | -9920.82 |
| Appropriateness ~ avatargender * avatarethnicity * area + Attractiveness_sc + avatargender:avatarethnicity:rwa_sc + (1 + avatargender + avatarethnicity + area \| ID) | 33 | 19912.12 | 20101.52 | -9923.06 |
| Appropriateness ~ avatargender * avatarethnicity * area + Attractiveness_sc + avatargender:avatarethnicity:sdo_sc + (1 + avatargender + avatarethnicity + area \| ID) | 33 | 19912.36 | 20101.76 | -9923.18 |
| Appropriateness ~ avatargender * avatarethnicity * area + stq_sc + (1 + avatargender + avatarethnicity + area \| ID) | 29 | 19912.46 | 20078.91 | -9927.23 |
| Appropriateness ~ avatargender * avatarethnicity * area + Attractiveness_sc + DistanceMean + (1 + avatargender + area \| ID) | 25 | 19915.45 | 20058.93 | -9932.72 |
| Appropriateness ~ avatargender * avatarethnicity * area + (1 + avatargender + avatarethnicity + area \| ID) | 28 | 19915.6 | 20076.31 | -9929.8 |

**Pleasantness of touch**

**Tab.S14** Model comparison for predictors of Pleasantness in heterosexual caucasian men. Candidate mixed-effects models were compared using maximum likelihood estimation and ranked by AIC. The best fitting model (lowest AIC) included the effects of Attractiveness.

| **Formula** | **npar** | **AIC** | **BIC** | **logLik** |
| --- | --- | --- | --- | --- |
| Pleasantness ~ avatargender * avatarethnicity * area + Attractiveness_sc + Trustworthiness_sc + (1 + avatargender + avatarethnicity + area \| ID) | 30 | 19694.43 | 19866.62 | -9817.21 |
| Pleasantness ~ avatargender * avatarethnicity * area + Attractiveness_sc + (1 + avatargender + avatarethnicity + area \| ID) | 29 | 19704.07 | 19870.52 | -9823.04 |
| Pleasantness ~ avatargender * avatarethnicity * area + Attractiveness_sc + avatargender:avatarethnicity:itg_sc + (1 + avatargender + avatarethnicity + area \| ID) | 29 | 19705.52 | 19894.93 | -9819.76 |
| Pleasantness ~ avatargender * avatarethnicity * area + Attractiveness_sc + avatargender:rwa_sc + (1 + avatargender + avatarethnicity + area \| ID) | 31 | 19707.23 | 19885.16 | -9822.61 |
| Pleasantness ~ avatargender * avatarethnicity * area + Attractiveness_sc + avatargender:sdo_sc + (1 + avatargender + avatarethnicity + area \| ID) | 31 | 19707.64 | 19885.58 | -9822.82 |
| Pleasantness ~ avatargender * avatarethnicity * area + Attractiveness_sc + avatargender:avatarethnicity:iat_sc + (1 + avatargender + avatarethnicity + area \| ID) | 33 | 19707.9 | 19897.31 | -9820.95 |
| Pleasantness ~ avatargender * avatarethnicity * area + Attractiveness_sc + avatarethnicity:area:itg_sc + (1 + avatargender + avatarethnicity + area \| ID) | 35 | 19708.4 | 19909.29 | -9819.2 |
| Pleasantness ~ avatargender * avatarethnicity * area + Attractiveness_sc + avatargender:avatarethnicity:sdo_sc + (1 + avatargender + avatarethnicity + area \| ID) | 33 | 19709.73 | 19899.14 | -9821.86 |
| Pleasantness ~ avatargender * avatarethnicity * area + Attractiveness_sc + avatargender:avatarethnicity:rwa_sc + (1 + avatargender + avatarethnicity + area \| ID) | 33 | 19710.09 | 19899.5 | -9822.05 |
| Pleasantness ~ avatargender * avatarethnicity * area + Attractiveness_sc + avatarethnicity:area:iat_sc + (1 + avatargender + avatarethnicity + area \| ID) | 35 | 19710.52 | 19911.41 | -9820.26 |
| Pleasantness ~ avatargender * avatarethnicity * area + Attractiveness_sc + avatarethnicity:area:sdo_sc + (1 + avatargender + avatarethnicity + area \| ID) | 35 | 19711.33 | 19912.23 | -9820.67 |
| Pleasantness ~ avatargender * avatarethnicity * area + Attractiveness_sc + avatarethnicity:area:rwa_sc + (1 + avatargender + avatarethnicity + area \| ID) | 35 | 19711.49 | 19912.39 | -9820.75 |
| Pleasantness ~ avatargender * avatarethnicity * area + Trustworthiness_sc + (1 + avatargender + avatarethnicity + area \| ID) | 29 | 19740.16 | 19906.62 | -9841.08 |
| Pleasantness ~ avatargender * avatarethnicity * area + stq_sc + (1 + avatargender + avatarethnicity + area \| ID) | 29 | 19765.95 | 19932.4 | -9853.98 |
| Pleasantness ~ avatargender * avatarethnicity * area + (1 + avatargender + avatarethnicity + area \| ID) | 28 | 19770.3 | 19931.01 | -9857.15 |

**Disgust of touch**

The linear mixed-effects model (R^2^marginal = .15, R^2^conditional = .62) for Disgust revealed a significant main effect of Avatar Gender (χ²(1) = 20.87, *p* < 0.001), with touches from male avatars being rated as significantly more disgusting than touches from female avatars (*b* = -6.56, *SE* = 1.42, *t* = -4.61, p < .001). A significant main effect of Area was also found (χ²(2) = 65.10, *p* < .001), indicating that intimate touches were rated as more disgusting than neutral (*b* = 15.3, *SE* 3.10, *t* = 4.92, *p* < .001) and social touches (*b* = 28.4, *SE* = 3.80, *t* = 7.47, *p* < .001), while social touches were rated as the least disgusting overall (b = -13.1, *SE* = 1.92, *t* = 6.83, *p* < .001). Additionally, a significant main effect of Attractiveness emerged (χ²(1) = 13.60, p < .001), suggesting that less attractive avatars were generally rated as more disgusting (b = - 2.91, *SE* = 0.79, t -3.69). An interaction between Avatar Gender and Avatar Ethnicity was also significant (χ²(1) = 6.16, *p* = .013), with pairwise comparisons revealing that African female avatars were rated as less disgusting than Caucasian female avatars (*b* = -3.22, *SE* = 1.48, *t* = -2.17, *p* = 0.032), while no significant difference was found among male avatars (*b* = 1.06, *SE* = 1.54, *t* = 0.69, *p* = .492). Finally, a significant three-way interaction between Avatar Gender, Avatar Ethnicity, and Area was detected (χ²(2) = 6.22, *p* = .045). Notably, in the neutral area, African female avatars were rated as less disgusting than Caucasian female avatars (*b* = = -5.58, *SE* = 2.22, *p* = .012), while no significant differences were found in the intimate or social areas.

**Tab.S15** Model comparison for predictors of Disgust in heterosexual caucasian women. Candidate mixed-effects models were compared using maximum likelihood estimation and ranked by AIC. The best fitting model (lowest AIC) included the effects of Attractiveness.

| **Formula** | **npar** | **AIC** | **BIC** | **logLik** |
| --- | --- | --- | --- | --- |
| Disgust ~ avatargender * avatarethnicity * area + Attractiveness_sc + Trustworthiness_sc + (1 + avatargender + avatarethnicity + area \| ID) | 30 | 20738.15 | 20910.35 | -10339.08 |
| Disgust ~ avatargender * avatarethnicity * area + Attractiveness_sc + (1 + avatargender + avatarethnicity + area \| ID) | 29 | 20741.45 | 20907.9 | -10341.72 |
| Disgust ~ avatargender * avatarethnicity * area + Trustworthiness_sc + (1 + avatargender + avatarethnicity + area \| ID) | 29 | 20743.37 | 20909.83 | -10342.69 |
| Disgust ~ avatargender * avatarethnicity * area + Attractiveness_sc + avatargender:sdo_sc + (1 + avatargender + avatarethnicity + area \| ID) | 31 | 20744.64 | 20922.57 | -10341.32 |
| Disgust ~ avatargender * avatarethnicity * area + Attractiveness_sc + avatargender:rwa_sc + (1 + avatargender + avatarethnicity + area \| ID) | 31 | 20744.83 | 20922.76 | -10341.42 |
| Disgust ~ avatargender * avatarethnicity * area + Attractiveness_sc + avatarethnicity:area:iat_sc + (1 + avatargender + avatarethnicity + area \| ID) | 35 | 20746.38 | 20947.27 | -10338.19 |
| Disgust ~ avatargender * avatarethnicity * area + Attractiveness_sc + avatargender:avatarethnicity:iat_sc + (1 + avatargender + avatarethnicity + area \| ID) | 33 | 20746.84 | 20936.25 | -10340.42 |
| Disgust ~ avatargender * avatarethnicity * area + Attractiveness_sc + avatargender:avatarethnicity:sdo_sc + (1 + avatargender + avatarethnicity + area \| ID) | 33 | 20747.18 | 20936.59 | -10340.59 |
| Disgust ~ avatargender * avatarethnicity * area + Attractiveness_sc + avatargender:avatarethnicity:rwa_sc + (1 + avatargender + avatarethnicity + area \| ID) | 33 | 20747.81 | 20937.22 | -10340.91 |
| Disgust ~ avatargender * avatarethnicity * area + Attractiveness_sc + avatargender:avatarethnicity:itg_sc + (1 + avatargender + avatarethnicity + area \| ID) | 33 | 20747.9 | 20937.32 | -10340.95 |
| Disgust ~ avatargender * avatarethnicity * area + Attractiveness_sc + avatarethnicity:area:itg_sc + (1 + avatargender + avatarethnicity + area \| ID) | 35 | 20750.53 | 20951.42 | -10340.26 |
| Disgust ~ avatargender * avatarethnicity * area + Attractiveness_sc + avatarethnicity:area:sdo_sc + (1 + avatargender + avatarethnicity + area \| ID) | 35 | 20751.49 | 20952.38 | -10340.74 |
| Disgust ~ avatargender * avatarethnicity * area + Attractiveness_sc + avatarethnicity:area:rwa_sc + (1 + avatargender + avatarethnicity + area \| ID) | 35 | 20752.17 | 20953.07 | -10341.09 |
| Disgust ~ avatargender * avatarethnicity * area + (1 + avatargender + avatarethnicity + area \| ID) | 28 | 20752.35 | 20913.06 | -10348.17 |
| Disgust ~ avatargender * avatarethnicity * area + stq_sc + (1 + avatargender + avatarethnicity + area \| ID) | 29 | 20753.21 | 20919.66 | -10347.61 |
|  |  |  |  |  |

**Erogeneity of touch**

**Tab.S16** Model comparison for predictors of Erogeneity in heterosexual caucasian women. Candidate mixed-effects models were compared using maximum likelihood estimation and ranked by AIC. The best fitting model (lowest AIC) included the effects of Attractiveness.

| **Formula** | **npar** | **AIC** | **BIC** | **logLik** |
| --- | --- | --- | --- | --- |
| Erogeneity ~ avatargender * avatarethnicity * area + Attractiveness_sc + (1 + avatargender + avatarethnicity + area \| ID) | 29 | 19843.83 | 20010.27 | -9892.92 |
| Erogeneity ~ avatargender * avatarethnicity * area + Attractiveness_sc + Trustworthiness_sc + (1 + avatargender + avatarethnicity + area \| ID) | 30 | 19843.97 | 20016.15 | -9891.99 |
| Erogeneity ~ avatargender * avatarethnicity * area + Attractiveness_sc + avatargender:avatarethnicity:iat_sc + (1 + avatargender + avatarethnicity + area \| ID) | 33 | 19844.91 | 20034.31 | -9889.46 |
| Erogeneity ~ avatargender * avatarethnicity * area + Attractiveness_sc + avatargender:avatarethnicity:itg_sc + (1 + avatargender + avatarethnicity + area \| ID) | 33 | 19845.13 | 20034.53 | -9889.56 |
| Erogeneity ~ avatargender * avatarethnicity * area + Attractiveness_sc + avatargender:sdo_sc + (1 + avatargender + avatarethnicity + area \| ID) | 31 | 19846.77 | 20024.69 | -9892.38 |
| Erogeneity ~ avatargender * avatarethnicity * area + Attractiveness_sc + avatargender:rwa_sc + (1 + avatargender + avatarethnicity + area \| ID) | 31 | 19847.15 | 20025.07 | -9892.58 |
| Erogeneity ~ avatargender * avatarethnicity * area + Attractiveness_sc + avatargender:avatarethnicity:rwa_sc + (1 + avatargender + avatarethnicity + area \| ID) | 33 | 19847.35 | 20036.75 | -9890.67 |
| Erogeneity ~ avatargender * avatarethnicity * area + Attractiveness_sc + avatargender:avatarethnicity:sdo_sc + (1 + avatargender + avatarethnicity + area \| ID) | 33 | 19847.55 | 20036.95 | -9890.77 |
| Erogeneity ~ avatargender * avatarethnicity * area + Trustworthiness_sc + (1 + avatargender + avatarethnicity + area \| ID) | 29 | 19898.68 | 20065.13 | -9920.34 |
| Erogeneity ~ avatargender * avatarethnicity * area + stq_sc + (1 + avatargender + avatarethnicity + area \| ID) | 29 | 19911.02 | 20077.46 | -9926.51 |
| Erogeneity ~ avatargender * avatarethnicity * area + (1 + avatargender + avatarethnicity + area \| ID) | 28 | 19912.47 | 20073.17 | -9928.23 |

**PHYSIOLOGICAL REACTIVITY TO TOUCH**

**Tab.S17** Model comparison for predictors of SCRs in heterosexual caucasian women. Candidate mixed-effects models were compared using maximum likelihood estimation and ranked by AIC. The best fitting model (lowest AIC) included the effect of STQ.

**Skin Conductance Responses**

| **Formula** | **npar** | **AIC** | **BIC** | **logLik** |
| --- | --- | --- | --- | --- |
| log_scr ~ avatargender * ethnicity * area + stq_sc + (1 + avatargender + ethnicity \| ID) | 20 | 5888.94 | 6003.75 | -2924.47 |
| log_scr ~ avatargender * ethnicity * area + stq_sc + avatargender:ethnicity:iat_sc + (1 + avatargender + ethnicity \| ID) | 24 | 5890.04 | 6027.81 | -2921.02 |
| log_scr ~ avatargender * ethnicity * area + stq_sc + avatargender:sdo_sc + (1 + avatargender + ethnicity \| ID) | 22 | 5892.58 | 6018.88 | -2924.29 |
| log_scr ~ avatargender * ethnicity * area + stq_sc + avatargender:ethnicity:itg_sc + (1 + avatargender + ethnicity \| ID) | 24 | 5892.68 | 6030.45 | -2922.34 |
| log_scr ~ avatargender * ethnicity * area + stq_sc + avatargender:rwa_sc + (1 + avatargender + ethnicity \| ID) | 22 | 5892.82 | 6019.12 | -2924.41 |
| log_scr ~ avatargender * ethnicity * area + stq_sc + avatargender:ethnicity:sdo_sc + (1 + avatargender + ethnicity \| ID) | 24 | 5895.38 | 6033.16 | -2923.69 |
| log_scr ~ avatargender * ethnicity * area + stq_sc + avatargender:ethnicity:rwa_sc + (1 + avatargender + ethnicity \| ID) | 24 | 5896.28 | 6034.06 | -2924.14 |
|  |  |  |  |  |

**Tab.S18** Model comparison for predictors of HR in heterosexual caucasian women. Candidate mixed-effects models were compared using maximum likelihood estimation and ranked by AIC. The best fitting model (lowest AIC) included the effect of Attractiveness and the interaction between Touching Avatar Ethnicity and SDO.

**Heart Rate**

| **Formula** | **npar** | **AIC** | **BIC** | **logLik** |
| --- | --- | --- | --- | --- |
| hr3 ~ avatargender * ethnicity * area + Attractiveness_sc + ethnicity:sdo_sc + (1 + avatargender \| ID) | 19 | 11357.05 | 11464.12 | -5659.53 |
| hr3 ~ avatargender * ethnicity * area + Attractiveness_sc + ethnicity:itg_sc + (1 + avatargender \| ID) | 19 | 11358.69 | 11465.76 | -5660.34 |
| hr3 ~ avatargender * ethnicity * area + Attractiveness_sc + ethnicity:rwa_sc + (1 + avatargender \| ID) | 19 | 11360.33 | 11467.4 | -5661.17 |
| hr3 ~ avatargender * ethnicity * area + Attractiveness_sc + ethnicity:avatargender:sdo_sc + (1 + avatargender \| ID) | 21 | 11360.89 | 11479.24 | -5659.45 |
| hr3 ~ avatargender * ethnicity * area + Attractiveness_sc + (1 + avatargender \| ID) | 17 | 11361.85 | 11457.65 | -5663.92 |
| hr3 ~ avatargender * ethnicity * area + Attractiveness_sc + avatargender:rwa_sc + (1 + avatargender \| ID) | 19 | 11361.93 | 11469 | -5661.96 |
| hr3 ~ avatargender * ethnicity * area + Attractiveness_sc + avatargender:ethnicity:itg_sc + (1 + avatargender \| ID) | 21 | 11362.38 | 11480.72 | -5660.19 |
| hr3 ~ avatargender * ethnicity * area + Attractiveness_sc + ethnicity:iat_sc + (1 + avatargender \| ID) | 19 | 11362.63 | 11469.7 | -5662.31 |
| hr3 ~ avatargender * ethnicity * area + Attractiveness_sc + avatargender:ethnicity:iat_sc + (1 + avatargender \| ID) | 21 | 11366.03 | 11484.37 | -5662.02 |

**SCL**

The model (R² marginal = 0.021. R² conditional = 0.021) did not show any significant modulation of this measure in relation to the avatar’s gender. avatarethnicity. or Attractiveness and Trustworthiness (all ps > .05). The interaction between avatar gender and avatarethnicity was also not significant (*p* = 0.69). suggesting that tonic skin conductance levels were not influenced by the experimental manipulations nor by individual differences in social touch attitudes.

**Heart Rate Variability**

None of the measures (RMSSD. HF. SampleEn) showed significant results.

**Interpersonal Distance**

**Tab.S19** Model comparison for predictors of IPD in heterosexual caucasian women.

| **Formula** | **npar** | **AIC** | **BIC** | **logLik** |
| --- | --- | --- | --- | --- |
| distance ~ avatargender * avatarethnicity + Attractiveness_sc + Trustworthiness_sc + avatargender:sdo_sc + (1 \| ID) | 10 | 1765.44 | 1798.01 | -872.72 |
| distance ~ avatargender * avatarethnicity + Attractiveness_sc + Trustworthiness_sc + avatargender:avatarethnicity:iat_sc + (1 \| ID) | 12 | 1766.53 | 1805.62 | -871.27 |
| distance ~ avatargender * avatarethnicity + Attractiveness_sc + Trustworthiness_sc + avatargender: rwa_sc + (1 \| ID) | 10 | 1766.73 | 1799.31 | -873.37 |
| distance ~ avatargender * avatarethnicity + Attractiveness_sc + Trustworthiness_sc + avatargender:avatarethnicity:itg_sc + (1 \| ID) | 12 | 1766.90 | 1805.99 | -871.45 |
| distance ~ avatargender * avatarethnicity + Attractiveness_sc + Trustworthiness_sc + avatargender:avatarethnicity:sdo_sc + (1 \| ID) | 12 | 1767.97 | 1807.06 | -871.99 |
| distance ~ avatargender * avatarethnicity + Attractiveness_sc + Trustworthiness_sc + (1 \| ID) | 7 | 1768.02 | 1790.83 | -877.01 |
| distance ~ avatargender * avatarethnicity + Attractiveness_sc + Trustworthiness_sc + avatargender:avatarethnicity:rwa_sc + (1 \| ID) | 12 | 1769.74 | 1808.83 | -872.87 |

**Body ownership questionnaire**

The linear mixed-effects model (R^2^marginal = 0.47. R^2^conditional = 0.92) revealed a significant main effect of the Question factor (χ²(1) = 93.63. *p* < 0.001). with higher ratings for the embodiment items (mean = 70. *SE* = 3.6) compared to the control items (mean 23. SE = 3.1 ; b = -46, *SE* = 4.8. *t* = -9.70. *p* < 0.001). suggesting that participants experienced a sense of body ownership over the virtual avatar. No other interactions reached significance.

**Vicarious touch**

The analysis of subjective reports concerning the illusory sensation of being touched on one’s own body (R²marginal = 0.011. R²conditional = 0.8) revealed a significant effect of the avatar’s gender (χ²(1) = 9.30. p = 0.0023). with a higher level of illusion during male touch (*b* = -6. *SE* 1.95. *t* = -3.050. *p* = 0.003) compared to female touch (mean for female-touching avatar = 55. *SE* = 4.1. vs. mean for male-touching avatar = 61. *SE* = 4.1). No other interactions reached significance.

**References**

Benedek. M.. & Kaernbach. C. (2010). A continuous measure of phasic electrodermal activity. *Journal of neuroscience methods*. *190*(1). 80-91.

Tarvainen. M. P.. Niskanen. J. P.. Lipponen. J. A.. Ranta-Aho. P. O.. & Karjalainen. P. A. (2014). Kubios HRV–heart rate variability analysis software. *Computer methods and programs in biomedicine*. 113(1). 210-220.
